# Nitric Oxide: Physiological Functions, Delivery, and Biomedical Applications

**DOI:** 10.1002/advs.202303259

**Published:** 2023-08-26

**Authors:** Syed Muntazir Andrabi, Navatha Shree Sharma, Anik Karan, S. M. Shatil Shahriar, Brent Cordon, Bing Ma, Jingwei Xie

**Affiliations:** ^1^ Department of Surgery‐Transplant and Mary & Dick Holland Regenerative Medicine Program College of Medicine University of Nebraska Medical Center Omaha NE 68198 USA; ^2^ Cell Therapy Manufacturing Facility MedStar Georgetown University Hospital Washington, DC 2007 USA; ^3^ Department of Mechanical and Materials Engineering College of Engineering University of Nebraska Lincoln Lincoln NE 68588 USA

**Keywords:** biomedical applications, delivery, donors, nitric oxide, physiological functions

## Abstract

Nitric oxide (NO) is a gaseous molecule that has a central role in signaling pathways involved in numerous physiological processes (e.g., vasodilation, neurotransmission, inflammation, apoptosis, and tumor growth). Due to its gaseous form, NO has a short half‐life, and its physiology role is concentration dependent, often restricting its function to a target site. Providing NO from an external source is beneficial in promoting cellular functions and treatment of different pathological conditions. Hence, the multifaceted role of NO in physiology and pathology has garnered massive interest in developing strategies to deliver exogenous NO for the treatment of various regenerative and biomedical complexities. NO‐releasing platforms or donors capable of delivering NO in a controlled and sustained manner to target tissues or organs have advanced in the past few decades. This review article discusses in detail the generation of NO via the enzymatic functions of NO synthase as well as from NO donors and the multiple biological and pathological processes that NO modulates. The methods for incorporating of NO donors into diverse biomaterials including physical, chemical, or supramolecular techniques are summarized. Then, these NO‐releasing platforms are highlighted in terms of advancing treatment strategies for various medical problems.

## Graphical abstract

This review explores nitric oxide's (NO) roles in physiology and pathology while considering its advantages and limitations. Progress in designing versatile materials as NO donors and carriers for bioavailability and function at target sites is discussed. It summarizes therapeutic potential, challenges, and prospects in biomedicine. It elaborates on developing dynamic NO‐releasing materials for tissue engineering and regenerative medicine, addressing critical concerns.

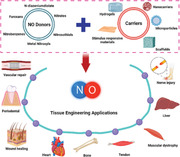

## 1 Introduction

**Figure 1:**
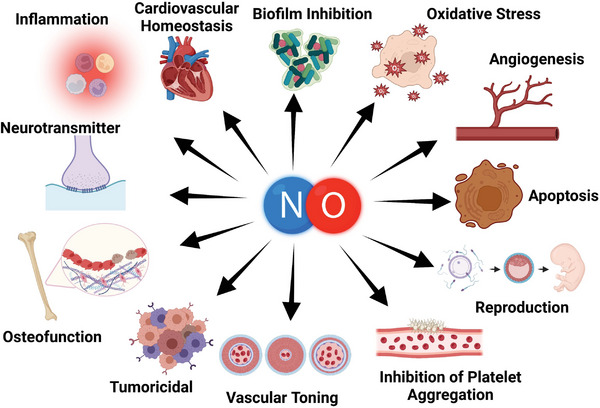
Major physiological roles mediated by exogenously and endogenously produced NO. Figure created using BioRender.com.

Nitric oxide (NO) is a ubiquitous gaseous molecule that is water soluble and can pass freely across cell membranes. It has a free radical structure making it notoriously noxious and possesses an extra electron which allows it to be highly reactive.^[^ ^1^ ^]^ In the late 1900s, NO was reported as an endothelial‐derived relaxing factor produced by blood vessels instrumental in vasodilation.^[^ ^2^ ^]^ Endogenous NO serves as an important effector and signal transduction molecule in numerous cellular processes involved in physiological states such as vasodilation, immune responses, neurotransmission, apoptosis, reproduction, regulation of gene transcription, mRNA translation, and post‐translational modifications of proteins.^[^ ^3^ ^]^ These physiological functions of NO are promoted at extremely low concentrations ranging from pico‐nanomolar.^[^ ^4^ ^]^ NO dysregulation occurs due to decreased synthesis, half‐life in tissues, and potency leading to cardiovascular diseases and aging. On the contrary, higher concentrations of NO promote oxidative stress as its cellular properties and targets are different leading to cytotoxicity. Under these circumstances, NO causes diseases related to neurotransmission and cancer.^[^ ^5^ ^]^ The role of NO in cellular processes and signaling has been well elucidated, which is mainly concentration dependent. As NO has a multifaceted role in physiologic and pathologic scenarios, NO must be delivered to a target site in the right dose at the right time to exert biological functions (**Figure** **1**). However, NO gas is quite challenging to handle as it prevents oxidation to nitrogen dioxide by readily excluding oxygen. Nevertheless, delivery of NO gas to the lungs via inhalation has proven to be therapeutic in pulmonary hypertension.^[^ ^6^ ^]^ Apart from this specific application and given the volatile nature of NO, a molecular carrier is often required to steadily release it at a target site. Due to the enormous therapeutic potential of NO, research in the past two decades has been focused on developing NO‐releasing platforms and donors that can precisely control the amount of NO released at target sites while limiting cytotoxicity. NO donors not only store and release NO but also improve NO pharmacokinetics. In this review article, we first detailed the process of NO production and its signaling, which is involved in numerous roles in human physiology and diseases. We next discussed various NO donors, delivery systems, and their applications in the biomedical field. Finally, we concluded and presented perspectives that can prompt future research in the field of NO.

## 2 NO Production, Signaling, and Roles in Human Physiology and Diseases

### 2.1 NO Production

Endogenous NO is enzymatically generated from l‐arginine by a distinct family of NO synthase (NOS). NOSs are oxidoreductase homodimeric enzymes constitutively expressed by neuronal cells (nNOS or NOS1), immune cells (inducible NOS, iNOS or NOS2) as well as endothelial cells (eNOS or NOS3).^[^ ^7^ ^]^ All isoforms of NOS oxidize and cleave the guanidine group of l‐arginine to release NO and yield l‐citrulline as a co‐product. NOSs utilize cofactors such as nicotinamide adenine dinucleotide phosphate (NADPH) as electron donors and require oxygen for the catalysis of l‐arginine.^[^ ^8^ ^]^ The rate‐limiting step in the NOS‐dependent release of NO is the availability of intracellular l‐arginine.^[^ ^9^ ^]^ The level of intracellular arginine is regulated by the action of arginosuccinate lyase, which converts citrulline back to arginine, thereby maintaining the levels of l‐arginine within the cell.^[^ ^10^ ^]^ NOS is composed of: i) an oxygenase domain at the amino‐terminal which contains the binding sites for l‐arginine and cofactor tetrahydrobiopterin (BH_4_), as well as a cluster of ferric heme and ii) a reductase domain that binds electron donors’ NADPH, flavin adenine dinucleotide (FAD), and flavin mononucleotide (FMN). The oxygenase and reductase domains are connected by a specific sequence that binds the Ca^2+^‐activated calmodulin complex.^[^ ^7^, ^11^ ^]^ Once NOS is activated, electrons from NADPH are transferred by the flavins in the reductase domain to the heme group in the oxygenase domain of the other monomer. This causes the binding of oxygen (O_2_) to the reduced heme iron (Fe^2+^) resulting in the sequential conversion of l‐arginine to HO‐l‐arginine, then NO and l‐citrulline.^[^ ^12^ ^]^ The transfer of electrons is enabled by the recruitment of the Ca^2+^‐calmodulin complex. High intracellular Ca^2+^ levels (with a half‐maximal activity between 200 and 400 nm) are crucial for this complex to bind eNOS and nNOS; while basal Ca^2+^ concentrations (below 40 nm) promote its binding in iNOS.^[^ ^13^ ^]^ All NOS isoforms have a zinc thiolate cluster formed by zinc coordination in a tetrahedral conformation with two cysteine (C) motifs (CXXXXC) at the dimer interface. Zinc in NOS is mainly for structural support rather than catalytic function.^[^ ^14^ ^]^ The zinc thiolate cluster is critical for the binding of BH_4_ and l‐arginine.^[^ ^14^, ^15^ ^]^ During the transfer of electrons from the reductase domain to the oxygenase domain, BH_4_ is oxidized to either a trihydrobiopterin radical (BH_3_^•^) or a trihydropterin radical cation protonated at the N5 position (BH_3_^•^H+). This radical cation can be reduced to BH_4_ either by NOS which utilizes the electron released by the flavins, or by reducing agents such as ascorbic acid which are present in the cells.^[^ ^7^, ^16^ ^]^ The NO released by NOS can target numerous enzymes and proteins in the cells. It mainly activates soluble guanylyl cyclase and generates cyclic guanosine monophosphate (cGMP).^[^ ^17^ ^]^ Each isoform is localized to a cellular‐subcellular location which subsequently dictates compartmentalized NO production and its downstream effector signaling.

eNOS is a 133 kDa protein with 1203 amino acids encoded by the NOS3 gene located on 7q35‐7q36 of human chromosome 7. It is expressed mainly in endothelial cells of the vascular endothelium and endocardium as well as cardiomyocytes, platelets, and erythrocytes.^[^ ^18^ ^]^ Activation of eNOS is induced by shear stress and strain. eNOS mRNA stabilization and protein expression are co‐operatively regulated by the transcriptional factors nuclear factor‐κB (NF‐κB) and Krüppel‐like factor 2 (KLF2).^[^ ^19^ ^]^ Another factor that influences the expression and activation of eNOS is reactive oxygen species. Specifically, hydrogen peroxide mediates eNOS transcription by either receptor‐mediated or physical stimulus leading to the activation of p38 mitogen‐activated protein kinase (MAPK). Other factors that can regulate the eNOS expression include epigenetic changes such as DNA methylation and polymorphisms that can influence its gene expression as well as numerous microRNA that regulate eNOS post‐transcriptionally.^[^ ^19b^ ^]^ eNOS activation is further fine‐tuned by post‐translational modifications such as phosphorylation, acetylation, *S*‐nitrosylation, and *S*‐glutathionylation.^[^ ^19^, ^20^ ^]^ In an inactive form, eNOS is acylated on glycine, cysteine, and lysine residues which enables its attachment to the transverse‐tubule plasma membrane, sarcolemma, and to a lesser extent to the Golgi apparatus in endothelial cells and cardiac myocytes. eNOS interacts with the scaffolding domain of caveolin 1 or 3 to remain in its inactive state.^[^ ^21^ ^]^ Upon ligand‐receptor binding or agonist stimulation, diacylation of eNOS causes its release from the membrane followed by an increase in the intracellular calcium levels to disrupt the eNOS‐caveolin complex. Recruitment of heat shock protein 90 and RACα serine/threonine protein kinase phosphorylates eNOS on serine residue 1177 (activation site) while protein phosphatases, PP1 and PP2A23 and PP2B (calcineurin) dephosphorylate Threonine 495 to block the inhibitory site.^[^ ^19^, ^22^ ^]^ Active eNOS translocate into the cytosol where additional kinases dictate its role in NO synthesis. Phosphorylation of eNOS at serine 615 or 633 by 5ʹ‐AMP‐activated protein kinase (AMPK), calcium/calmodulin‐dependent protein kinase type II (CaMKII) and cAMP‐dependent protein kinase (PKA) and tyrosine 81 by protooncogene tyrosine‐protein kinase Src results in increased NO production.^[^ ^19b^ ^]^ On the other hand, phosphorylation of serine 114 by ERK1, ERK2, and protein kinase C and tyrosine 657 by protein‐tyrosine kinase 2β (PYK2) is associated with a decrease in NO synthesis.^[^ ^23^ ^]^ S‐nitrosylation of zinc tetrathiolate cluster at cysteine 94 and 99 residues limits electro‐transfer with the homodimer leading to eNOS inhibition and agonist stimulation of eNOS causes denitrosylation.^[^ ^20c^ ^]^ eNOS uncoupling occurs in pro‐oxidative conditions due to S‐glutathionylation.^[^ ^20d^ ^]^

nNOS is a 1434 amino acid‐containing protein with a molecular weight of 161 KDa encoded by the NOS1 gene present on the 12q24.2 location on chromosome 12 in humans. It is highly expressed in vascular smooth muscle cells (VMSC), synaptic spines, sarcoplasmic reticulum of cardiomyocytes, cardiac neurons, and ganglia.^[^ ^21^, ^22^ ^]^ nNOS share a structural similarity to the other NOS except for the presence of a PDZ (post‐synaptic density protein, discs‐large, ZO‐1) domain at its N‐terminus. nNOS utilizes the PDZ domain to interact with other PDZ‐containing proteins.^[^ ^22d^ ^]^ Five splice variants of nNOS are reported to date: nNOSα, nNOSβ, nNOSµ, nNOSγ, and nNOS1‐2. Of these, only nNOSα and nNOSµ have PDZ domain which by interaction with other proteins, can restrict the localization of nNOSα and nNOSµ to the plasma membrane connected via α‐syntrophin in myocytes.^[^ ^23^ ^]^ nNOSβ and nNOSγ do not have a PDZ domain and are present in the cytosol.^[^ ^18b^ ^]^ Depending on the anchoring of nNOS to the membrane or cytosolic proteins via PDZ‐PDZ or C‐terminal‐PDZ interaction, NO activity varies. nNOS activation is promoted by post‐synaptic density protein‐95 (PSD95) that can couple N‐methyl‐d‐aspartate receptor (NMDAR) with nNOS leading to NO release.^[^ ^24^ ^]^ Another adaptor protein, the carboxyl‐terminal PDZ ligand of neuronal NOS protein (CAPON) binds to the N‐terminal PDZ domain of nNOS to enhance nNOS activity. CAPON interacts with Dexras1 (a brain‐enriched member of the Ras family of small G proteins) which is selectively induced by dexamethasone.^[^ ^25^ ^]^ The interaction of nNOS and CAPON can deliver released NO to Dexras1 to promote its S‐nitrosylation at cysteine 11 which is important in iron uptake by neurons.

Three nNOS phosphorylations have been reported to date. Phosphorylation of Ser 852 in a CaMKII‐dependent manner reduces the activity of nNOS by blocking the binding of calcium‐calmodulin. On the contrary, another phosphorylation of Ser 1417 in an AKT‐dependent manner elevates the nNOS function in cardiac cells and VMSCs.^[^ ^19^, ^26^ ^]^ Phosphorylation of nNOS at Ser1412 by AMPK is observed in murine HL‐1 cardiomyocytes which are exposed to oxidative stress.^[^ ^27^ ^]^ The function of nNOS is regulated by its compartmentalization to the myocardium through modulating calcium fluxes in the intracellular or sarcolemma space at the sarcoplasmic reticulum and plasma membrane respectively.^[^ ^28^ ^]^ The nNOS function is inhibited in all tissues other than the myocardium by the interaction of endogenous inhibitor, and protein inhibitor of nNOS (PIN).^[^ ^29^ ^]^ It can interfere with electron transfer from the reductase domain to the oxygenase domain along with making the heme centers inaccessible to oxygen.

*iNOS*: Earlier studies on NO identified its pro‐inflammatory functions in macrophages but it is clear from recent studies that NO is released and mediated by different cells involved in anti‐inflammation.^[^ ^30^ ^]^ The dual inflammatory response of NO is mainly based on the cells involved and the complex chemistry of the NO functions. In inflammatory processes, NO is mainly released by the enzymatic function of iNOS which is not typically expressed in resting cells and is induced by the inflammatory cytokines or microbes.^[^ ^30b,c^ ^]^ iNOS is a 1153 amino acid protein with a molecular mass of 130 kDa. It is encoded by the *NOS2* gene located on human chromosome 17 at 17q11.2‐q12. Expression of iNOS occurs primarily in oxidative or inflammatory conditions in various cells such as macrophages, VMSCs, fibroblasts, endothelial cells, cardiomyocytes, leukocytes, and nerve cells.^[^ ^19^, ^31^ ^]^ It is located in the cytosol to produce NO. In comparison to eNOS and nNOS, it is calcium‐dependent, and its expression levels are transcriptionally regulated.^[^ ^32^ ^]^ iNOS remains highly stable at both the mRNA and protein levels.^[^ ^30a^ ^]^ Once assembled, it can function 100–1000 higher than that of eNOS and nNOS under basal levels of intracellular calcium. With high activity, iNOS can generate large amounts of NO for a longer duration (days) until either the levels of substrates and cofactors decrease or the degradation of the enzyme itself.^[^ ^33^ ^]^ The sustained high release of NO will cause the production of various reactive nitrogen oxide species (RNOS). These RNOS can mediate a large spectrum of physiological and pathological remodeling functions occurring in the cardiovascular system.^[^ ^34^ ^]^ Hence, the release and function of iNOS need to be regulated. In an inflammatory response, activated macrophages are central effector cells. They can release pro‐inflammatory cytokines such as tumor necrosis factor (TNF‐*α*) and interleukin‐1*β* (IL‐1*β*) or immunomodulatory/anti‐inflammatory cytokines such as IL‐4, IL‐6, IL‐10, and transforming growth factor β (TGF‐β). The levels of pro‐inflammatory and anti‐inflammatory cytokines regulate the levels of NO. An increase in NO release is promoted by the excessive production of pro‐inflammatory cytokines while in the presence of anti‐inflammatory cytokines, the release of NO is suppressed by downregulating the expression of iNOS.^[^ ^30^, ^35^ ^]^

### 2.2 NO Signaling

NO is structurally simple as well as highly reactive and can readily form various nitrogen oxides which result in a decrease in its bioavailability. It has a very short half‐life under physiological conditions. It can only travel very limited distances before being oxidized. Reaction products of NO such as nitrite, nitrate, and derivative of S‐ or N‐nitroso proteins and iron‐nitrosyl complexes are not only metabolic products but can be reduced to release NO by the means of numerous reactions. These NO‐containing products not only function as reservoirs for NO but can also travel to remote tissues via circulation to make NO available for recipient tissues.^[^ ^36^ ^]^ Hence, the bioavailability of NO is dependent both on free NO radicals and NO‐releasing compounds. Additionally, oxidizing agents present in the cytoplasm of cells can also limit the intracellular bioactivity of NO by reducing the diffusion efficiency of NO (which is normally within ≈100 µm from its origin). The limited diffusion of NO combined with the NOS subcellular localization confines NO functions to target proteins that are co‐localized with NOS within multiprotein signalosomes.^[^ ^4^ ^]^

NO signaling works via classical and non‐classical mechanisms to promote cellular functions. The classical NO signaling mechanism has a long range and the signal is transmitted relatively long distances from the NO source.^[^ ^37^ ^]^ The classical mechanism of NO signaling is achieved through the activation of guanylate cyclase. NO binds to the prosthetic heme group on the enzyme to activate its soluble form which can, in turn, catalyze the conversion of guanosine 5′‐triphosphate (GTP) to 3′,5′‐cyclic guanosine monophosphate (cGMP). cGMP is the second messenger molecule and activates cGMP‐dependent serine/threonine protein kinase (PKG) which then modulates various cell processes including cardio‐protection (from both reactive hypertrophy and reperfusion injury), inflammatory responses, phagocytic defense mechanisms, inhibition of platelet aggregation, vasodilation, neurotransmission, and calcium homeostasis.^[^ ^38^ ^]^ cGMP is hydrolyzed into an inactive 5′‐GMP metabolite by the phosphodiesterase enzyme. A balance between the levels of soluble guanylate cyclase and inhibitory phosphodiesterase determines the levels of cGMP. The rate of cGMP synthesis is 10‐fold lower than its catabolic conversion by PDE in most cells.^[^ ^39^ ^]^

The non‐classical mechanism of NO signaling involves the covalent post‐translational modification of biomolecules by NO and its derivatives. S‐nitrosylation of protein thiols, oxidative nitration, hydroxylation, and metal nitrosylation of transition metals are the most common modifications promoted by NO.^[^ ^40^ ^]^ Nitrosylation occurs by the covalent incorporation of an NO nitrosyl moiety into another molecule. Nitrosylation at the thiol group of cysteine is named S‐nitrosylation, while nitrosylation of a transition metal is referred to as metal nitrosylation. S‐nitrosylation occurs at physiological pH and is a known mechanism to regulate protein conformational changes, and post‐translational modifications such as phosphorylation, acetylation, ubiquitination, methylation, disulfide bond formation, and hydroxylation.^[^ ^40^, ^41^ ^]^ S‐nitrosylation regulates cell processes such as transcription, DNA repair, growth, differentiation, and apoptosis.^[^ ^40^, ^41^ ^]^ In metal nitrosylation, NO interacts with the metal center of a heme molecule to activate or inhibit the function of proteins. For instance, NO binds to the ferrous heme of soluble guanylate cyclase (sGC) causing a conformational change that activates it. On the other hand, when NO binds to the heme of cytochrome C, which is involved in the electron transport chain located within the mitochondria, the function of cytochrome C is blocked.^[^ ^42^ ^]^ NO also has a higher binding affinity to ferrous hemoglobin than oxygen or carbon dioxide. In ischemia‐reperfusion where oxygen levels are elevated, hemoglobin preferentially binds to NO which displaces oxygen and confers protection to the tissues from oxygen toxicity.^[^ ^43^ ^]^ NO has protective effects at pico‐ and nanomolar concentrations. However, it is cytotoxic at higher concentrations. It can react with reactive oxygen species (ROS), specifically superoxide, to form peroxynitrate causing peroxidation of lipids, thiols, amines, fatty acids, nitrate tyrosine and hydroxylate guanines at low pH. These conditions lead to oxidative/nitrosative stress which causes the release of anti‐inflammatory signals.^[^ ^42b^ ^]^ The non‐canonical pathway is short‐ranged and occurs at subcellular locations close to the NO source.^[^ ^44^ ^]^ The transnitrosylation reaction is a process by which a nitrosylated protein (either at the cysteine group (S‐nitrosylation) or the metal center of a heme group (metal nitrosylation)) transfers its nitrosyl moiety to an interacting protein containing a cysteine thiol motif (I/L‐X‐C‐X2‐D/E consensus).^[^ ^45^ ^]^ This reaction occurs successively to increase the transmission range of an NO signal from its source to its various subcellular target locations.^[^ ^45^, ^46^ ^]^

NO can be inactivated by reacting with superoxide anion (O_2_^−•^) to form oxidant peroxynitrite (ONOO^−^) which is highly potent to cells as it causes nitrosative and oxidative stress leading to S‐nitrosylation of biomolecules such as proteins, lipids, and DNA as well as nitration.^[^ ^47^ ^]^ It also causes DNA single‐strand breaks, resulting in the activation of poly‐ADP‐ribose polymerase (PARP), which directs the fate of the cell (DNA repair or cell death) based on the type and extent of the stimulus.^[^ ^48^ ^]^

### 2.3 Role in the Vascular System

One of the first physiological functions discovered for NO was its ability to act as a vasodilator in the cardiovascular system. Since this discovery, studies have revealed various roles of NO in many different physiological and pathological functions, such as infection, inflammation, fetal and postnatal development, angiogenesis, hypertrophy, and programmed cell death (apoptosis). This review article first discusses the diverse roles of NO in cardiovascular functions.

The role of NO in the regulation of vascular tone was observed nearly four decades ago.^[^ ^49^ ^]^ The activation of eNOS, which is expressed by endothelial cells, is the initial step in the classical signaling pathway leading to vasodilation. Inactive eNOS is normally bound to caveolin located in cell membrane invaginations called caveolae.^[^ ^50^ ^]^ eNOS activation occurs in a sequential manner. It is activated by either the release of intracellular calcium reserves from the endoplasmic reticulum (ER); or by the opening of voltage‐dependent Ca^2+^ channels, which allows extracellular Ca^2+^ to enter the cell and increase the Ca^2+^ levels of the cytosol. Ca^2+^ binds calmodulin which undergoes conformational changes to enable its binding to eNOS in the caveolae. eNOS is then released leading to its activation.^[^ ^51^ ^]^ Once in the cytosol, eNOS converts l‐arginine to NO. NO regulates vasorelaxation through three different signaling pathways. ***i)*** It diffuses into the adjacent vascular smooth muscle cells (VSMC) and activates sGC to induce cGMP release. cGMP then activates PKG which can block the calcium influx from the voltage‐dependent calcium channels as well as calcium release from the ER, the process of which is normally mediated by inositol 1,4,5‐trisphosphate receptor (IP_3_R).^[^ ^52^ ^]^ PKG also upregulates the calcium ATPase pump (SERCA) present on the ER to enable the uptake of calcium from the cytosol.^[^ ^53^ ^]^ These actions cause the levels of intracellular Ca^2+^ to drop, which in turn inactivates calmodulin. The inactivation of calmodulin deactivates myosin light chain kinase (MLCK). Meanwhile, low calcium levels activate myosin light chain phosphatase (MLCP) resulting in the breakage of actin‐myosin cross‐bridges. This event causes the VSMC to relax.^[^ ^54^ ^]^ ***ii)*** Under hypoxic conditions NO‐induced sGC produces cyclic inosine 3′,5′‐monophosphate (cIMP) instead of cGMP. cIMP activates Rho‐associated protein kinase (ROCK) which blocks MLCP thereby promoting the contraction of VSMC.^[^ ^55^ ^]^ ***iii)*** NO promotes nitrosothiol formation via S‐nitrosylation which can induce long‐lasting relaxation of VSMC. S‐nitrosylation elevates the function of SERCA, this increases the uptake of cytoplasmic Ca^2+^ stores into the ER.^[^ ^53^, ^56^ ^]^ Impairment of the NO‐sGC‐cGMP signaling pathway is reported to prompt endothelial dysfunction leading to hypertension, atherosclerosis, heart stroke, and failure.^[^ ^57^ ^]^ Research in this direction has revealed multiple processes that contribute to aberrant vascular toning which includes lower NO generation due to reduced NOS function, accelerated degradation of cGMP, and downregulation of signaling targets of cGMP.^[^ ^56^, ^58^ ^]^ However, endothelial dysfunction is not solely a result of decreased NO production by eNOS, but rather a combination of factors including reduced l‐arginine availability, deregulated enzymatic function, and a high rate of NO degradation.^[^ ^59^ ^]^ Hence, futuristic studies should consider the bioavailability of NO and its sources, as well as the various complex signaling pathways in which it is involved when assessing the role of NO in vascular wall toning.

### 2.4 Inhibition of Platelet Aggregation

The process of hemostasis involves the coordinated functions of platelets, coagulation factors, and endothelial cells that line the blood vessels. The vascular tone and hemostasis in the blood vessels are maintained by the interactions between the vessel wall, platelets, and platelet‐derived signaling factors involved in vaso‐activation and aggregation. In a normal blood vessel, the endothelial cells lining the vessel resist interaction with platelets and coagulation factors to prevent thrombosis. A vessel wall injury occurring either by endothelial denudation (superficial injury) or plaque rupture (deep intimal injury) exposes collagen and von Willebrand factor.^[^ ^60^ ^]^ This leads to the initiation of a series of events that promote the closure of the vessel wall injury. The first step is the activation of the platelets to form a thrombus. Platelets are activated by tissue‐factor‐mediated thrombin generation and exposed collagen, which promotes thrombus formation and recruits additional platelets by releasing platelet‐derived factors such as ADP, thromboxane, and serotonin.^[^ ^61^ ^]^ Activated platelets show changes in shape, surface receptor expression, attachment, and aggregation; these changes lead to the formation of a thrombus. The activation and recruitment of platelets to the site of injury are tightly regulated as hyper‐aggregation of platelets can contribute to increased thrombosis and thereby impair cardiovascular functions. A balance between pro‐ and anti‐aggregation stimuli is maintained by tuning platelet function to achieve normal hemostasis. Under physiological conditions, anti‐aggregatory factors limit the functions of pro‐aggregatory signals. In normal conditions, anti‐aggregatory autacoids produced by endothelial cells, such as NO and prostacyclin prevent the attachment of platelets to the endothelial wall lining. NO is reported to be a negative regulator of thrombosis as it inhibits platelet activation and aggregation.^[^ ^62^ ^]^ It is predominantly released from l‐arginine by eNOS. Apart from endothelial cells, platelets also release NO in small amounts by the enzymatic action of eNOS (predominant) and iNOS.^[^ ^63^ ^]^ Platelets produce NO in resting and stimulated conditions. Platelet‐derived NO acts in a feedback mechanism to regulate platelet activation in an autocrine manner.^[^ ^64^ ^]^ Exogenous NO inhibits the expression of platelet surface glycoproteins such as p‐selectin and the integrin glycoprotein IIB/IIIa complex both of which are normally upregulated during platelet activation. NO (via cGMP released by the sGC) can stimulate cGMP‐ dependent kinase to reduce fibrinogen binding to glycoprotein IIb/IIIa, as well as modulate responses mediated by phospholipase A2 and C to inhibit platelet aggregation. Additionally, NO promotes the dissociation of fibrinogen from glycoprotein IIb/IIIa by inhibiting the platelet cytosolic Ca^2+^ in a cGMP‐ dependent manner and attenuating phosphoinositol‐3‐kinase function.^[^ ^60a‐c^ ^]^ However, cardiovascular pathological conditions such as acute coronary syndrome, diabetes, chronic heart failure, atrial fibrillation, coronary artery spasm, and stable angina pectoris can cause an imbalance between pro‐ and antiaggregatory stimuli leading to hyper platelet aggregation and activation.^[^ ^60a^ ^]^ Evidence indicates resistance to NO by sGC due to oxidation of its heme moiety under oxidative stress.^[^ ^19^, ^65^ ^]^ It should be noted that dysregulated NOS function is often observed in most cardiovascular diseases, leading to a decline in NO concentrations.^[^ ^7a^ ^]^ It is noteworthy to investigate whether these ailments are a result of decreased NO synthesis, NO resistance, or a combination of both. Insights from such research can facilitate the development of more effective treatment strategies in the future.

### 2.5 Inflammation and Anti‐inflammation

NO functions as a pro‐inflammatory, as well as an anti‐inflammatory molecule, and its levels and site of release are tightly regulated. Physiological levels of NO favor anti‐inflammation. During the onset of inflammation, circulating neutrophils reach the inflamed site by moving across the endothelium from the blood via chemotaxis. Following neutrophils, monocytes also move to the site of injury. Then, due to the release of cytokines, monocytes differentiate into macrophages. Macrophages then phagocytose damaged cells present at the site of injury/inflammation. Pro‐inflammatory cytokines induce the expression of iNOS in macrophages, neutrophils, and granulocytes, as do endotoxins released by bacterial infections. Activated iNOS promotes the release of large quantities of NO (a 1000‐fold increase) to fight inflammation.^[^ ^30b^ ^]^ The released NO protects blood vessels from endogenous injury and interferes with early and late conduit vessel atherogenesis.^[^ ^66^ ^]^ It delays endothelial cell death by decreasing pro‐inflammatory signals. It inhibits the adhesion and migration of neutrophils and monocytes by decreasing surface adhesion molecules such as p‐selectin, CD11, and CD18.^[^ ^67^ ^]^ Depending on the location and cell type, NO production favors either inflammation or anti‐inflammation. Low levels of NO released by eNOS stimulate pro‐inflammatory cytokines such as cyclooxygenase 2 and nuclear factor kappa B (NFκB).^[^ ^68^ ^]^ Elevated levels of eNOS reduce oxidative stress, inflammation, and renal damage which can occur during the process of renal ischemia‐reperfusion; while increased iNOS induces damage and inflammation.^[^ ^69^ ^]^ It is evident that levels of iNOS increase during inflammation, asthma, infection, and stimulation of the immune system.^[^ ^70^ ^]^ Factors such as cyclooxygenase, tumor necrosis factor α, interleukin‐1β, lipopolysaccharide, interferon‐γ, as well as NFκB, all play a role in elevating the function of iNOS during inflammation.^[^ ^71^ ^]^ Hence, downregulating the expression of iNOS using inhibitors, such as glucocorticoids, can reduce inflammatory responses.^[^ ^72^ ^]^ A study revealed that the fumagillin prodrug, released by Aspergillus fumigatus, can induce endothelial NO production.^[^ ^73^ ^]^ This, in turn, activates autophagy through the AMP‐activated protein kinase (AMPK)/mammalian target of the rapamycin (mTOR) signaling pathway. Activation of autophagic machinery suppressed NF‐kB signaling thereby downregulating cytokine release associated with inflammation.

While most studies have primarily focused on the immune responses associated with iNOS‐generated NO, it is worth noting that nNOS and eNOS are also involved in NO release and metabolism. However, the immunological role of these variants still requires elucidation. Additionally, further in‐depth studies are needed to identify the NO signaling cascade, the mechanism of NO action, and their specific targets. Such investigations will provide valuable insights for the development of therapeutic approaches.

### 2.6 Oxidative Stress

The induction of oxidative stress is mainly associated with pathological conditions such as ischemia‐reperfusion injury, dilated cardiomyopathy, atherothrombosis, vascular dysfunction, and heart failure. It is critical to restore redox balance to treat these conditions.^[^ ^74^ ^]^ The redox potential of the tissues is normally regulated but changes drastically in pathological scenarios. When oxygen accepts an electron, it becomes a ROS that is highly reactive in the presence of free radicals containing one or more unpaired electrons such as superoxide anion, hydroxyl radical, hydrogen peroxide, lipid peroxide, and hypochlorous acid. Being a free radical molecule, NO reacts with superoxide to form reactive nitrogen species (RNS). Under physiological conditions, cells protect themselves from ROS via an antioxidant release. An imbalance between the oxidants and antioxidants leads to oxidative stress.^[^ ^58^, ^75^ ^]^ Additionally, the electrons from the electron transport chain, present in mitochondria and necessary for energy production, are uncoupled during the electron transfer (normally completed via cytochrome c oxidase), leading to superoxide formation.^[^ ^58^ ^]^ Uncoupling of eNOS occurs due to deficiencies of substrates (l‐arginine and oxygen) and BH_4_ co‐factor causing ROS production.^[^ ^76^ ^]^ When the levels of superoxide increase, it can react with itself to form H_2_O_2_ and O_2_ by spontaneous enzymatic dismutation reactions.^[^ ^75^, ^77^ ^]^ Additionally, H_2_O_2 (_via metal‐catalyzed Fenton reaction), forms highly reactive hydroxyl radicals which are strong oxidizing agents and can react with amino acids, carbohydrates, lipids, and nucleic acids to modify them.^[^ ^78^ ^]^ In the physiological system, NO prevents the formation of reactive hydroxyl radicals by limiting the Fenton reaction. However, hydroxyl radical‐induced endothelial injury suppresses the production and function of NO. This is because NO is oxidized to nitrite and nitrate which cannot promote vasodilation.^[^ ^79^ ^]^

NO has pro‐oxidative as well as anti‐oxidative functions in lipid peroxidation.^[^ ^80^ ^]^ NO is inactivated by superoxide to release peroxynitrite, a highly potent oxidant.^[^ ^80^, ^81^ ^]^ In atherosclerosis, oxidation of low‐density lipoproteins (LDL) by peroxynitrite (released by resident endothelial cells) promotes monocyte recruitment and foam cell formation.^[^ ^82^ ^]^ On the other hand, antioxidant effects of NO are observed at high concentrations, which leads to the inhibition of LDL oxidation crucial in the initiation and propagation of vascular lesions.^[^ ^83^ ^]^ The net effect of NO on lipid peroxidation is determined by the levels of NO and superoxide which are simultaneously released by endothelial cells. An excess of NO inhibits lipid peroxidation while either an excess of superoxide or equimolar concentrations of NO and superoxide induces lipid peroxidation.^[^ ^80^ ^]^ Apart from inhibiting LDL oxidation, NO can also scavenge ROS directly.^[^ ^84^ ^]^

### 2.7 Angiogenic Potential

NO plays multiple roles in modulating angiogenesis. It acts as an endothelial cell survival factor as it not only functions to inhibit apoptosis but also promotes proliferation via increasing the expression of vascular endothelial growth factor (VEGF) and fibroblast growth factor (FGF).^[^ ^85^ ^]^ NO enhances the migration of endothelial cells through the stimulation of podokinesis.^[^ ^86^ ^]^ It also promotes the expression of α_v_β_3_ and degrades the extracellular matrix (ECM) by inducing the FGF to upregulate urokinase‐type plasminogen activator.^[^ ^87^ ^]^ NO is also reported to mediate tumor vascularization and blood flow.^[^ ^88^ ^]^ eNOS from endothelial cells plays a central role in angiogenesis. In the past few decades, numerous studies have reported angiogenic and antiangiogenic properties of NO. One possible explanation for this discrepancy is that the concentration of NO and duration of exposure determine its function.^[^ ^88b^ ^]^ Signaling molecules such as protein kinase C (PKC) phosphorylation, extracellular‐signal‐regulated protein kinase (ERK), Jun, and activation of activator protein 1 (AP1) are promoted by NO at low concentrations but inhibited at higher concentrations.^[^ ^89^ ^]^ NO modulates the functions of angiogenic and physiological factors such as VEGF, shear stress, estrogen, angiopoietin, sphingosine‐1‐phosphate, and oxidative and metabolic stress. These modulators activate the eNOS necessary for the release of NO. Specifically, VEGF‐mediated angiogenesis and vascular permeability function via eNOS. VEGF promotes the upregulation of eNOS expression by increasing levels of mRNA and protein synthesis.^[^ ^90^ ^]^ eNOS is activated by its phosphorylation at Ser615, Tyr81, Ser633, and Ser1177 via the functions of multiple kinases. VEGFR2 signaling has a downstream effect in endothelial cells, namely the activation of eNOS by phosphorylation at Ser1177 in an Akt‐dependent manner. Shear stress also activates eNOS via Akt and PKA signaling.^[^ ^88b^ ^]^ NO released by the enzymatic function of eNOS activates sGC‐cGMP‐PKG downstream signaling to promote angiogenesis. NO also upregulates matrix metalloprotease 2 (MMP‐2) in the endothelium, while simultaneously downregulating tissue inhibitors of metalloprotease‐1 and −2. In addition, eNOS mediates prostaglandin E_2_ (PGE_2_) to activate the PI‐3K/Akt pathway to promote endothelial cell sprouting via NO/cGMP signaling.^[^ ^88b^ ^]^

### 2.8 Modulation of Apoptosis

Apoptosis is programmed cell death in which cells are killed in a highly regulated manner. Stimuli such as activation of death receptors (e.g., Fas/TRAIL), as well as cellular stress from DNA damage, infection, and inflammation lead to the release of cytochrome C from mitochondria, thereby activating apoptotic machinery and signaling. NO can induce or inhibit the process of apoptosis in a wide variety of cells based on the cell type and the amount of NO present. The cytotoxicity of NO released by iNOS and nNOS is primarily due to its ability to interact with superoxide to form peroxynitrite, thereby making NO proapoptotic.^[^ ^91^ ^]^ Peroxynitrite formation is determined by the ratio of NO to superoxide levels; the susceptibility of cells to peroxynitrite is dependent on the levels of antioxidants present in the cell. Peroxynitrite and elevated levels of NO cause DNA damage initiating apoptosis of cells (e.g., macrophages, islets of the pancreas, thymocytes, and specific neurons) as DNA damage leads to the accumulation of p53, a tumor suppressor protein that upregulates p21 to induce cell cycle arrest.^[^ ^91b^ ^]^ In addition, NO stimulates sGC to promote apoptosis in VSMCs.

Despite the cytotoxic effector functions of NO, one study showed an antiapoptotic role of NO in human B lymphocytes which enriched the understanding of NO's functions in apoptosis.^[^ ^92^ ^]^ Further research revealed that NO inhibits apoptosis in different cell types (e.g., leukocytes, hepatocytes, trophoblasts, and endothelial cells).^[^ ^91a^ ^]^ NO regulates apoptotic signaling at multiple stages. Nitrosylation of the active site of caspases by NO results in inhibition of caspase's activity.^[^ ^93^ ^]^ NO regulates death‐inducing signaling complex (DISC) formation, leading to reduced cleavage of Bid, thereby inhibiting the amplification of apoptotic signals through the mitochondria.^[^ ^94^ ^]^ NO also modulates the expression of death receptors in a cGMP‐dependent manner and alters the expression of acid sphingomyelinase, which in turn reduces DISC formation.^[^ ^95^ ^]^ Additionally, NO upregulates Bcl‐2 and Bcl‐XL to inhibit cytochrome C release from mitochondria.^[^ ^92^ ^]^

### 2.9 Role of NO in the Heart

The stimulation of the β‐adrenergic receptor by the sympathetic nervous system is a potent physiological response mechanism that regulates cardiac function. It plays a role in central nerve functions, peripheral blood circulation, cardiac muscle contraction, metabolic regulation, and heart rate.^[^ ^96^ ^]^ Acute β‐adrenergic stimulation controls cardiac output during stress or physical exercise, commonly referred to as the “fight or flight” response. Conversely, chronic β‐adrenergic stimulation is crucial in both physiological and pathological cardiac remodeling.^[^ ^97^ ^]^ NO is crucial for the β‐adrenergic mediated functions of the heart. In response to β‐adrenergic stimulation, nNOS bound to the ryanodine receptors in the sarcoplasmic reticulum (SR) of cardiomyocytes, increases the NO levels in these cells.^[^ ^98^ ^]^ To further aid in β‐adrenergic stimulation, NO signals the release of calcium from the SR through S‐nitrosylation of calcium/calmodulin‐dependent protein kinase II and the ryanodine receptor, leading to their activation. nNOS is critical in the release of calcium from the SR.^[^ ^99^ ^]^ Inhibition of nNOS by peroxynitrite and superoxide reduces β‐adrenergic stimulation in cardiomyocytes and subsequently inhibits calcium release.^[^ ^100^ ^]^ Additionally, a decrease in NO accompanied by an increase in superoxide levels, along with blocking phospholamban phosphorylation, causes uncoupling of nNOS.^[^ ^101^ ^]^ Peroxynitrite alters the cardiomyocyte action potentials, increases lipid peroxidation which damages mitochondria, and suppresses cardiac muscle function via the SERCA.^[^ ^98^ ^]^ Elevated levels of peroxynitrite promote calcium sequestration through SERCA and relax the cardiomyocyte.^[^ ^102^ ^]^ nNOS can also be cardio‐protective during exercise by increasing the consumption of oxygen.^[^ ^98^ ^]^ eNOS inhibits the β‐adrenergic response by regulating the l‐type calcium channel which contributes to cardio‐protection.^[^ ^103^ ^]^ During exercise, the ratio of eNOS dimer to monomer increases while the formation rate of peroxynitrite decreases. Thus, decreased peroxynitrite levels increase eNOS dimerization and activation which protects the heart.^[^ ^104^ ^]^ iNOS primarily functions in the induction of inflammatory responses in the heart, as well as the development of reactive hypertrophy. During exercise, the levels of iNOS are low while in reactive hypertrophy elevated levels of iNOS are promoted by various signaling mechanisms. Increases in ROS, activation of PKB and ERK, and decreased PI3K levels activate iNOS.^[^ ^105^ ^]^ Elevated levels of NO increase mitochondrial oxygen consumption, within the cells of the heart, mitochondrial respiration is regulated by angiotensin II which modulates mitochondrial NOS.^[^ ^106^ ^]^ Cardiomyocytes function by controlling the diffusion and compartmentalization of NO. As NO is locally released via the function of NOS, heme‐centered proteins such as myoglobin and cytoglobin expressed by cardiomyocytes scavenge the released NO thereby regulating its diffusion in the heart.^[^ ^107^ ^]^

### 2.10 NO as Neurotransmitter

Neurotransmission plays a fundamental role in transferring information between neurons and their target cells, regulating numerous processes in the body.^[^ ^108^ ^]^ It facilitates both excitatory and inhibitory actions within the central nervous system, while also controlling autonomic and motor responses in the body. Neurotransmission occurs at specific junctions called synapses, which connect the presynaptic neuron to the postsynaptic target cell, enabling the relay of information. This intricate process involves the repeated exocytosis of synaptic vesicles containing neurotransmitters from the presynaptic neurons, followed by endocytosis of these vesicles into the postsynaptic terminal of the target cell. Such a process drives cognitive functions, including learning and memory, in the brain. NO is released by both pre‐and postsynaptic nerve endings. NO functions as an anterograde neurotransmitter at the presynaptic end of peripheral nitrergic nerves. It starts with the arrival of an action potential at the presynaptic ending which opens the voltage‐gated calcium channels to release calcium. The calcium then activates nNOS, which is important in various neuronal functions. There are nitrergic interneurons in the substantia gelatinosa of Rolando which regulate pain transmission between the nociceptive primary and secondary neurons in the spinal cord. nNOS controls functions of the nitrergic and enteric neurons of the gastrointestinal system.^[^ ^109^ ^]^ NO also activates the N‐methyl‐d‐aspartate receptors (NMDAR) to induce filopodial growth of the presynaptic nerve endings as well as regulate presynaptic plasticity in GABAergic and glutamatergic neurons.^[^ ^110^ ^]^

NO functions as a retrograde neurotransmitter involved in the activation of NMDAR of the postsynaptic hippocampal glutamatergic synapses. NMDAR is activated by the stimulation that comes from the binding of glutamate and glycine along with the release of magnesium from the top of the channel to allow the entry of sodium (Na^+^) and calcium to depolarize the membrane of the postsynaptic ending. Calcium activates Ca^2+^/CaM‐dependent kinase II to phosphorylate the glutamatergic α‐amino‐3‐hydroxy‐5‐methyl‐4‐isoxazole propionic acid (AMPA) receptor which increases the entry of calcium. Increased calcium activates nNOS to release NO. Calcium‐activated CaM kinase IV promotes phosphorylation of the transcription factor cAMP‐response element binding protein (CREB) to transcribe gene expression related to learning and memory.^[^ ^111^ ^]^ NO is released from the post‐synaptic terminal and diffuses across the synaptic cleft to the presynaptic terminal. NO then stimulates the release of vesicle‐bound neurotransmitters from the presynaptic terminal in a GC‐independent manner. NO also stimulates auto‐phosphorylating CaM kinases within the presynaptic terminal to further induce sustained signaling.^[^ ^111^, ^112^ ^]^

In addition, NO has a role in the activation of a transcription factor called eukaryotic initiation factor 2α (eIF2α) located in the dendritic spines. This takes place through the binding of NO to the heme group of heme‐regulated eIF2α kinase (HRI).^[^ ^113^ ^]^ The HRI inhibits the translation of most mRNAs under conditions of stress.^[^ ^114^ ^]^ NO induces phosphorylation of eIF2α to promote the translation of mRNAs that contain AUG (uAUG) in the 5′ untranslated region (5′UTR) as well as poly‐AUG mRNAs present in the granules of the stress bodies at the base of the dendritic spine.^[^ ^111b^ ^]^

### 2.11 Anti‐infection Property

Endogenous release of NO is critical in defending against infection. NO possesses antibacterial, antifungal, and antiviral properties making it a formidable molecule in the treatment of infectious diseases.

#### 2.11.1 Antibacterial Function

The bactericidal role of NO is mainly mediated by the chemical alteration of DNA. Enhanced oxidative stress and high NO levels increase the likelihood of interactions between oxygen and NO. These interactions increase ROS production leading to the formation of RNOS which include ONOO^–^ (peroxynitrite), NO_2_^•^, N_2_O_3,_ and other species. were also These secondary reaction products are responsible for the nitrosation, oxidation, and nitration of biomolecules that NO itself cannot interact with or alter.^[^ ^115^ ^]^ NO damages the DNA of bacteria via three mechanisms: direct DNA structural alteration by RNOS, blocking DNA repair, and increasing the generation of genotoxic alkylating agents and hydrogen peroxide.^[^ ^116^ ^]^ RNOS specifically N_2_O_3_ promotes the deamination of cytosine, adenine, and guanine residues present in the DNA resulting in DNA breaks. RNOS also interacts with and modifies proteins at cysteine, tyrosine, methionine, phenylalanine, and tryptophan residues.^[^ ^115a^ ^]^ The peroxynitrite free radical induces DNA breaks and mutations by removing purine and pyrimidine bases and creating basic sites along with lipid peroxidation of liposomes.^[^ ^116^, ^117^ ^]^ DNA repair is also hindered by the presence of NO. It inhibits the function of DNA alkyl transferases by reacting with the ‐SH group of cysteine residues to form NO adducts, which in turn inhibit the transfer of the alkyl group from guanine to the protein.^[^ ^118^ ^]^

Nitrosylation of thiols is an important NO‐mediated mechanism of cytotoxicity against microbes. Potent nitrosylators such as S‐nitrosothiols (RSNOs), N_2_O_3,_ and dinitrosyl‐thiol‐iron complexes are taken up readily by microbes. This alters microbial protein functions causing RSNOs‐mediated inhibition of Bacillus cereus spores.^[^ ^119^ ^]^ NO binds to heme‐containing proteins such as guanylate cyclase, cytochrome P450, and NOS. At low concentrations of RNOS, NO binds with the heme moiety of these proteins to modulate their functions. At higher concentrations of RNOS, NO irreversibly binds to heme‐containing proteins thereby removing the heme group from the protein. NO reduces Fe (III) complexes to promote hydroxyl radical formation causing the release of iron from metalloenzymes, which in turn results in the depletion of iron from bacteria.^[^ ^120^ ^]^ NO enables host defenses by not only combating microbes present in the respiratory tract but also by protecting the host from oxidative injury. For instance, NO protects against hydrogen peroxide‐mediated cytotoxicity in mammalian cells, while it enables the cytotoxic functions of hydrogen peroxide in *E. coli* as microbes are more sensitive to NO treatment. Bacterial iron‐sulfur clusters are central to many bacterial functions. Degradation of these clusters by NO or RNOS causes the release of iron, which catalyzes the formation of free radicals. These highly reactive free radicals bind to DNA, leading to DNA breaks along with cell membrane damage. The ability to penetrate the cell wall and subsequent promotion of nitrosative and oxidative reactions make NO a potent inhibitor of microbes. Microbial resistance to NO therapy was tested against *S. aureus*, methicillin‐resistant *S. aureus* (MRSA), *Staphylococcus epidermidis*, *E. coli*, and *Pseudomonas aeruginosa* and no significant increase in the minimum inhibitory concentration (MIC) was observed in these species. The inability of resistance development is because of diverse and multiple antimicrobial mechanisms exhibited by NO and may require several simultaneous mutations for microbial survival.^[^ ^115a^ ^]^

#### 2.11.2 Antiviral Properties

Like the antimicrobial function, NO can act as an antiviral agent using different modes of action. These include inhibition of viral enzymes via nitrosylation, promotion of oxidative and nitrosative stress to damage viral DNA, regulation of viral transcription factors that promote virulence, and activation of host defensive pathways.^[^ ^121^ ^]^ NO is cytotoxic against both DNA and RNA viruses. NO reacts with oxygen, superoxide, and hydrogen peroxide to generate RNOS such as peroxynitrite, which can bind thiol moieties on cysteine residues of viral and host proteins.^[^ ^122^ ^]^ NO enables host clearance via nitrosylation reactions on viral enzymes such as protease, reductase and reverse transcriptase. This blocks viral replication, transcription, and infectivity of virions such as human papillomavirus (HPV).^[^ ^123^ ^]^ Peroxynitrite impairs the viability of virions and has a modest inhibitory effect on viral replication in host cells.^[^ ^124^ ^]^ However, peroxynitrite does not discriminate between the host and viral DNA/RNA but with the robust repair machinery in place, the host tolerates and repairs the damage caused by peroxynitrite.^[^ ^125^ ^]^ If the endogenous production of NO is inadequate or inhibited, exogenous NO is required to attain an antiviral effect. For instance, in human rhinovirus infections, the endogenous levels of NO by iNOS are limited by feedback inhibition via NF‐κB and interferon regulatory factor 1 (IRF‐1) dependent transcription. But the exogenous release of NO still provides an antiviral effect.^[^ ^126^ ^]^ In addition, antiviral agents such as acetylsalicylic acid, chebulagic acid, and punicalagin suppress iNOS function via IKK‐NF‐κB and P38‐MAPK signaling pathways.^[^ ^121^, ^127^ ^]^ In some cases, viruses such as severe acute respiratory syndrome coronavirus 2 (SARS‐CoV‐2) use their membrane protein to suppress NF‐κB activity by binding with the inhibitor of NF‐κB kinase subunit β, which further implicates the need for an exogenous supply of NO via therapeutics.^[^ ^128^ ^]^

#### 2.11.3 Antifungal Activity

Most fungal infections are caused by pathogenic fungi and are superficial and cutaneous, limited to hair, nails, and epidermis. The most prevalent of these infections are dermatophytosis, candidiasis, and pityriasis versicolor. Candidiasis is caused by *Candida* fungi, of which the most frequent is *Candida albicans* (*C. albicans*). Most fungal infections are not life‐threatening but frequent occurrences can cause complications in immunocompromised patients. The function of NO in fungal infections is multifaceted. NO production in *C. albicans* is enabled by a NOS‐like enzyme that can catalyze the conversion of l‐arginine to NO and citrulline.^[^ ^129^ ^]^ NO protects the fungi from oxidative stress and azoles. The low endogenous levels of NO in *C. albicans* act in a feedback protection mechanism in response to oxidative stress.^[^ ^129a^ ^]^ In contrast, the exogenous release of NO kills *C. albicans* via nitrosative stress.^[^ ^129b^ ^]^ *C. albicans* were also resistant to host NO, which may result from the increased expression of either NOS inhibitors or the NO‐scavenging protein CaHYB1 released by *C. albicans*.^[^ ^130^ ^]^ Recent reports have shown that eNOS, but not iNOS, is involved in fungal innate immunity.^[^ ^131^ ^]^

### 2.12 Osteofunction

Bone is mainly comprised of osteoblasts, osteocytes, and osteoclasts that work in a highly regulated manner to maintain bone homeostasis. Osteoblasts, derived from mesenchymal stem cells, are responsible for building bone by laying down the bone matrix, which is crucial for maintaining bone architecture, strength, and rigidity. On the other hand, osteoclasts originated from hematopoietic stem cells found in bone marrow. These cells break down bone to promote bone remodeling in healthy bones and contribute to excessive bone loss in pathological conditions. In addition to osteoblasts and osteoclasts, osteocytes play a vital role. These are differentiated osteoblasts that become embedded in the bone matrix and release growth factors and signaling molecules to regulate the functions of both osteoblasts and osteoclasts. NO is a key messenger in bone homeostasis and pathology. Osteoblasts and osteoclasts produce NO to maintain bone homeostasis in response to various stimuli, such as proinflammatory cytokines, mechanical stimulation, shear stress, estrogen, thyroid hormone, and aging.^[^ ^132^ ^]^ All of the NOS isoforms are expressed in bone cells and of these eNOS is the most predominant; it is expressed in bone marrow mesenchymal cells (BM‐MSCs), osteoblasts, osteocytes, and osteoclasts. NO generated via eNOS is stimulated by estrogen alone and is critical for the functioning of osteoblasts.^[^ ^133^ ^]^ nNOS is reported to regulate bone mass and turnover in mice bone cell cultures.^[^ ^134^ ^]^ iNOS is initially present in fetal bone and its expression is decreased in adult bone. However, it is activated under inflammatory conditions.^[^ ^135^ ^]^ Early reports showed that arginosuccinate lysate (ASL) generates arginine from arginosuccinate which provides a substrate for NOS to produce NO in osteoblasts, while caveolin 1 inhibits NOS. NO production can also be enhanced by the coordinated action of ASL and cationic amino acid transporter 1, which transports arginine across the cell membrane.^[^ ^136^ ^]^ Intracellular NO induces aerobic glycolysis in pre and differentiated osteoblasts, while exogenously released NO from osteoblasts and osteocytes prevent osteoclast attachment and function.^[^ ^132^, ^136^ ^]^ NO has biphasic effects on osteoblasts and osteoclasts. At low concentrations, NO promotes IL‐1‐induced osteolytic function and stimulates osteoblast growth and differentiation. However, higher levels of NO inhibit osteoclast formation and osteogenesis, thereby promoting apoptosis in their respective progenitors.^[^ ^132a^ ^]^

### 2.13 NO in Cancer

NO exerts dichotomous effects in cancer initiation, progression, and metastasis. At nano to picomolar concentrations, it regulates various cellular processes including angiogenesis, apoptosis, cell cycle, invasion, and metastatic progression.^[^ ^137^ ^]^ NO promotes cell transformation by activating RNOS to mediate DNA breaks and mutations while simultaneously inhibiting DNA repair.^[^ ^138^ ^]^ iNOS‐induced NO causes GC to AT substitution mutations in p53 a tumor suppressor gene leading to its inactivation. NO inhibits apoptosis by blocking caspase activity, increasing Bcl‐2 expression, inducing heat‐shock proteins HSP70 and HSP32, suppressing the release of cytochrome C, and decreasing cyclooxygenase‐2 activation.^[^ ^138^ ^]^ NO also promotes arteriolar dilation which increases blood flow to the tumor, thereby inducing the angiogenesis necessary for tumor progression. In addition, NO upregulates MMP‐2 and MMP‐9 and downregulates inhibitors of MMP, tissue inhibitor matrix metalloprotease (TIMP) −2 and −3 to promote tumor invasion.^[^ ^139^ ^]^

Contrary to its tumor‐promoting functions, NO also exhibits tumoricidal properties when present at micromolar levels. NO produced by macrophages, natural killer cells, endothelial cells, and kuffer cells has a cytostatic/cytotoxic effect on tumor cells via regulating the functions of aconitase and ribonucleotide reductase.^[^ ^140^ ^]^ NO suppresses tumor DNA synthesis via *the* salvage pathway. Higher concentrations of NO become proapoptotic by activating the caspase cascade via the release of cytochrome C from mitochondria into the cytosol and by increasing p53 expression.^[^ ^141^ ^]^ NO either promotes or inhibits tumor growth based on its concentration, the type of cell present, redox potential, and exposure time in the tumor microenvironment. NO is pro‐tumorigenic at low concentrations and anti‐tumorigenic at higher concentrations (which are difficult to obtain within tumor cells unless NO donors are utilized exogenously). For a comprehensive understanding of NO's functions in cancer, please refer to the relevant review articles.^[^ ^142^ ^]^ Given its involvement in cancer biology, targeting aberrant NO signaling at various stages of carcinogenesis holds significant potential. However, a thorough understanding of the complex NO signaling in cancer is necessary. Future studies involving NO alone or in combination with anticancer drugs may unveil new venues for cancer treatment.

### 2.14 NO Function in Reproduction

NO plays a crucial role in all stages of mammalian reproduction. Its expression varies at different stages of development and maintenance in both female and male reproductive systems.^[^ ^143^ ^]^ In female reproduction, NO affects various processes, including follicle development, regulation of menstruation, oocyte maturation, ovulation, fertilization, embryogenesis, and maintenance of pregnancy until childbirth.^[^ ^144^ ^]^ In male reproduction, NO regulates sperm motility, hyperactivation, capacitation, and maturation.^[^ ^144^, ^145^ ^]^ These functions are orchestrated by the combined action of all three NOS enzymes expressed in reproducible organs, Leydig cells, Sertoli cells, spermatocytes, immature sperm head, smooth muscle, and endothelial cells, generating NO and activating NO‐sGC‐cGMP signaling.^[^ ^143^, ^144^ ^]^ Specifically, NO promotes the synthesis and secretion of follicle‐stimulating hormone, progesterone, estrogen, luteinizing hormone, gonadotrophin, and prostaglandin involved in reproduction.^[^ ^143^ ^]^ During follicle development, NO modulates estrogen levels and is believed to be a crucial player in tissue remodeling during ovulation and luteinization.^[^ ^146^ ^]^ It also regulates follicle angiogenesis to ensure a continuous supply of nutrients and oxygen to the developing follicle.^[^ ^147^ ^]^ Additionally, NO influences follicular atresia, the process of selecting dominant follicles that mature during ovulation.^[^ ^146^ ^]^ NO modulates the estrus cycle by regulating the survival and apoptosis of granulosa cells in the follicle after ovulation.^[^ ^148^ ^]^ In oocyte maturation, NO derived from iNOS controls the rupture of germinal vesicles and release of the first polar body, promoting meiosis and oocyte maturation in mouse models.^[^ ^149^ ^]^ Furthermore, NO is speculated to improve sperm capacitation in a time‐based concentration‐dependent manner.^[^ ^150^ ^]^ Low levels of NO can promote sperm motility, while high levels can reduce it.^[^ ^151^ ^]^

Studies have demonstrated that the early stages of embryo development are crucial processes susceptible to various stress factors, including inflammation and angiogenesis.^[^ ^152^ ^]^ NO plays an essential role in repairing damage by promoting the expression of genes associated with the repair mechanism.^[^ ^153^ ^]^ Additionally, NO is involved in embryo implantation by regulating the balance between cAMP/cGMP. Normal embryo development relies on NO to promote cGMP release, which modulates the cAMP/cGMP ratios.^[^ ^154^ ^]^ By exerting relaxation effects on smooth muscle, inhibiting platelet aggregation, and reducing inflammation, NO helps create a healthy uterine microenvironment by influencing the functions of steroid hormones and cytokine present in the endometrium and decidua.^[^ ^155^ ^]^ Moreover, it vasodilates umbilical arteries, facilitating uterine‐placental blood perfusion.^[^ ^144a^ ^]^ Insufficient levels of NO can lead to fetal abnormalities. However, during the onset of delivery, NO levels decline as NOS activity decreases in the uterus. Conversely, NO production increases in the pelvis to facilitate the opening of the pelvis during delivery.^[^ ^156^ ^]^ While many functions of NO in reproduction have been extensively studied, numerous questions remain unanswered. The mechanisms by which NO exerts its cellular‐level functions and induces physical changes in the body remain unclear. Additionally, why NO levels remain low to enable sperm motility is an intriguing question. Investigating the status and function of NO in conditions such as infertility, preeclampsia, rupture of uterine membrane, intrauterine growth restriction, and ectopic pregnancy, which are critical issues in the field of reproduction, would be worthwhile. For a detailed understanding of NO in reproduction, refer to the review by Luo et al.^[^ ^143^ ^]^ Figure 1 illustrates the different physiological roles of NO.

## 3 NO Donors and Carriers

**Figure 2:**
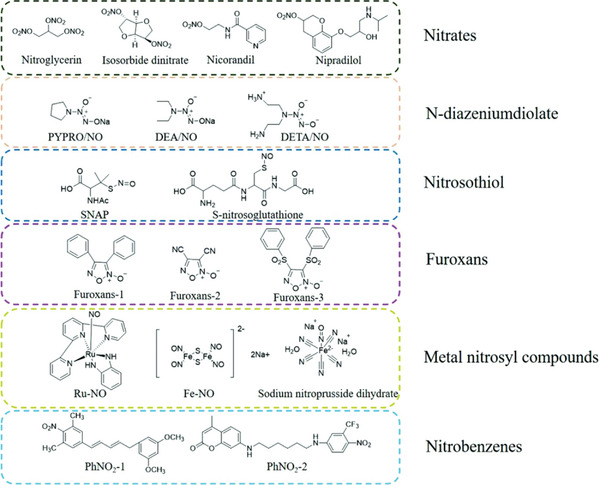
Schematic representation showing the structure of the several classes of NO donors categorized into specific groups. Reproduced with permission.^[^ ^173b^ ^]^ Copyright 2021, under Creative Commons Attribution License (CC BY).

Despite its diverse physiological roles, the therapeutic applications of NO remain restricted due to its short half‐life and insufficient concentrations of NO available on‐site. To overcome these challenges, advanced strategies are being employed to develop efficient NO‐generating systems involving multiple types of NO‐releasing carriers/donors. NO donors can be classified into several categories according to their nature and mechanism of NO release (**Figure** **2**). While all NO donors are considered pharmacologically active in physiological environments, it is not necessarily crucial for every donor to directly produce or generate NO for targeted delivery.^[^ ^4^ ^]^ Diazeniumdiolates (also referred to as NONOates) and RSNOs are the two main classes commonly used.^[^ ^157^ ^]^ The NONOates and RSNOs are well‐established NO donors, with NONOates capable of spontaneously generating two NO molecules from a single diazeniumdiolate residue. RSNO serves as natural NO reservoirs and carriers within biological systems.^[^ ^158^ ^]^ These small NO donors have improved the NO stability, biodistribution, and delivery. However, they still face significant challenges such as toxicity, storage problems due to photochemical and temperature instability, difficulty in regulating prolonged release, and resistance to certain compounds.^[^ ^158^, ^159^ ^]^ To overcome these issues, the use of macromolecular NO donors has shown promise in improving stability, biodistribution, transport across membranes, and circulation time, thereby establishing a safer and more efficient NO therapy. The mode of action and therapeutic efficacy may vary between donors based on their type, structure, and function. Several approaches including functionalization of materials, surface modification, and the use of stimuli response advanced scaffolds have been explored and used as promising NO donors (**Table** **1**). These stimuli‐responsive intelligent delivery vehicles release NO as a response to specific biological, chemical, and physical signals (e.g., enzymes, pH levels, temperature, light, and heat). In addition to modifications in the structure and functional groups, NO donors have been designed in such a way that they can react with acids, thiols, and glutathione to improve the stability and release profiles of NO.^[^ ^160^ ^]^ While new strategies for the development of promising NO donors/carriers continue to emerge, several obstacles still hinder the use of NO‐based therapies in clinical settings. These obstacles include concentration‐dependent biological effects and the stability of NO donors/carriers under physiological conditions. As a result, researchers are motivated to provide their critical insights in designing and developing advanced NO donor molecules for targeted and effective therapeutic applications.

**Table 1:** The table lists various NO donors, carriers, and possible outcomes. Modified and reproduced with permission.^[^ ^173b^ ^]^

NO carrier/delivery system	NO donor	NO payload	NO release half‐life or duration	Model systems	Outcome	References
Poly (lactic‐co‐glycolic acid)‐polyethylenimine‐ based NO releasing NPs	NONOate	0.04 µm NO mg^−1^ NPs,	≈6 days	In vivo; Mouse	NPs showed extended NO release and exhibited promising in‐vivo antibacterial activity against MRSA and *P. aeruginosa*. Enhanced wound healing	[167]
Gentamicin‐ conjugated POEGMA‐b‐PVBA NPs	Spermine NONOate	Not reported	75% NO release after ≈ 5 h	In vitro	Polymer conjugated Gen‐NO co‐delivery system for NO storing and release of NO and gentamicin. Co‐delivery NP effectively reduced P. *aeruginosa* biofilm	[225]
Injectable capric acid and octadecane encapsulated NONOate microparticles	In situ self‐assembling NO‐releasing micelle deposits	30 µm NONOate	t_1/2_ = 1298.3 ± 205.8 s w/Hb at 37 °C	In vivo; Ovariectomized rats with osteoporosis	Longer NO‐released in OVX‐induced osteoporosis rats reversing effects	[268]
Sol‐gel derived Silica‐NO NPs	GSNO	∼300 µm GSNO release	Duration of NO release >24 h	In vitro; Human clinical isolates	NO‐NP can generate and maintain GSNO formation over a prolonged time, where lower NO concentrations are more effective antimicrobial agents	[269]
Poly lactic‐co‐glycolic acid (PLGA) microspheres	SNAP	0.56(± 0.01) µm SNAP mg^−1^ microspheres	Uncapped SNAP = 10 days. PLGA encapsulated SNAP = ≈4 weeks	In vitro	Stable PLGA encapsulated SNAP microspheres for targeted NO release for a prolonged duration	[166]
Chitosan‐graft‐poly(amidoamine) dendrimer conjugated methicillin and NO (CS‐PAMAM‐MET/NONOate)	NONOate	4.42 µm mg^−1^	After 5 h, ≈80% of NO was released and continued over 16 h	In vivo; Rats	A co‐delivery system for NO and MET release for synergistic antibacterial effect against MRSA. Accelerated healing in infectious wounds	[174b]
NO‐releasing poly(amidoamine) dendrimers	Diazeniumdiolate	∼1 µm mg^−1^ total NO release	t_1/2_ ∼ 1 h	In vitro, Mouse fibroblasts	Size and exterior functionality are crucial in dendrimer‐bacteria association, NO delivery efficiency, bacteria membrane disruption, migration of biofilm, and mammalian toxicity	[194a]
Liposome‐encapsulated N‐diazeniumdiolates	Diazeniumdiolates	PROLI/NO ≈5.10 µm mg^−1^; DEA/NO ≈9.16 µm mg^−1^; PAPA/NO ≈8.86 µm mg^−1^; SPER/NO ≈7.73 µM mg^−1^	t_1/2_ in minute: PROLI/NO ≈ 9.6; DEA/NO ≈18.6; PAPA/NO ≈156; SPER/NO ≈2718	In vitro	NO release for an enhanced period. pH‐controlled release of NO for potential application against tumors	[182a]
S1P/JS‐K/Liposome	diazen‐1‐ium‐1,2‐diolate	Not reported	Without GSH, and GST storage conditions: 12% in 8 h. Under GSH, and GST storage conditions: >90% in 8 h	In vivo; Mice	Targeted release of NO for treating brain glioma tumors	[177]
Chitosan‐derived hydrogel/glass composite	Topical NO	2.5 mg mL^−1^ of NO NPs: initial peak 37.5 nm, steady state 20 nm; 5 mg mL^−1^ of NO NPs: initial peak 75 nm, steady state 50 nm; 10 mg mL^−1^ of NO‐NP: initial peak 150 nm, steady state 100 nm; 20 mg mL^−1^ of NO NPs: initial peak 300 nm, steady state 200 nm	Not measured	In vivo; Mice	Reduced suppurative infection, decreased microbial burden, reduced collagen degradation	[270]
Direct application of DNICs into wounds	Dinitrosyl iron complexes (DNIC‐1 and DNIC‐2)	Angiogenesis: 7.8 µm DNIC‐1, DNIC‐2 Diabetic hindlimb ischemia: 0.18 mg kg^−1^ DNIC	DNIC‐1 t_1/2_ = 27.4 ± 0.5 h at 25 °C and 16.8 ± 1.8 h at 37 °C DNIC‐2 t_1/2_ = 1.7 ± 0.1 h at 25 °C and 0.8 ± 0.1 h at 37 °C	In vivo; Mice	DNIC‐1 displays the best pro‐angiogenesis and restores impaired angiogenesis in ischemic hind limbs, increasing wound repair in diabetic mice	[271]
3D bone scaffolds	SNAP	10 wt% SNAP, initial NO release rate 0.5 ± 0.06 × 10^−10^ mol min^−1^ mg^−1^, NO release rate 0.23 ± 0.02 × 10^−10^	Theoretical t_1/2_ extrapolated to 8.6 days	In vivo; Mice fibroblasts	Reduction in *S*. *aureus* and *P*. *aeruginosa* adherence	[272]
Collagen matrix/DNIC‐GS composite spongy sheets	Dinitrosyl iron complexes with glutathione (DNIC‐ GS)	4.0 µm DNIC‐GS	Complete NO release from the matrix in 1 h after submersion in distilled water	In vivo; Rats	Enhanced growth, maturation, and fibrous transformation of granulation tissue	[273]
CS/NO releasing film	GSNO	0.625 mm GSNO (2.5, 10 and 20 wt%) in 20 g of chitosan solution	Ongoing NO release at 48 h for all concentrations	In vitro; Rats	Increased wound healing and epithelialization compared to chitosan‐only films	[274]
CS/NO releasing film	GSNO	0.26 µm NO mg^−1^ film	Continued NO release up to day 3	In vitro; Mice	Enhanced antibacterial activity against MRSA; Greater anti‐biofilm activity; Faster biofilm dispersal, wound size reduction, epithelialization rates, and collagen deposition than untreated and chitosan‐only groups	[275]
Xerogel‐coated glass slides	NONOate	Total NO released, µm cm^−2^: 3.3 ± 0.4, 2.5 ± 0.6, 2.6 ± 0.3, 1.9 ± 0.3, 2.3 ± 0.3 (0, 6, 12, 18, 24 coating layers, respectively)	Apparent t_1/2_, h: 11.4 ± 0.7, 13.6 ± 1.4, 17.8 ± 4.3, 13.2 ± 0.6, 16.3 ± 2.4 (0, 6, 12, 18, 24 coating layers, respectively)	In vitro; Mice fibroblasts	Reduction in *P*. *aeruginosa* compared to control	[276]
Gel	Sodium Nitrite	14.6 mm sodium nitrite mixed in equal amounts with low pH acid gel	The concentration of NO was maintained at 10 mm for >1 h after application	In vivo; Mice	Increased re‐epithelization by 50%, hair follicle regeneration, angiogenesis, procollagen—expressing fibroblasts, promotion, and infiltration of inflammatory cells in wound beds	[277]
Cream	Sodium Nitrite	3% (w/v) sodium nitrite mixed in equal amounts with 4.5% (w/v) citric acid in aqueous cream		In vivo; Human clinical trial	Acidified topical nitrites can clear 60% of MRSA wounds	[278]
NO‐releasing chitosan matrix	CBC‐NONOate	200 mg CBC‐NO (releases 250 nm NO per 5 mg of CBC‐NO)	≈180 min	In vivo; Rats	Day 17 post‐fracture: 20% increase in cross‐sectional area fracture callus compared to control; 30% compared to NOS inhibition	[173b]
Ruthenium‐based boronic acid‐decorated photosensitizer (PS)	Ruthenium‐based light‐triggered nitric oxide (RBNO) releasing agent	40 µm	Not measured	In vitro	Dispersal of biofilms and frees *P*. *aeruginosa* from the biofilm structure RBNO eradicate mature biofilms at low NO concentrations	[279]

### 3.1 NO Donating Polymeric Nanoparticles

The use of polymeric nanoparticles (NPs) as NO donors is highly advantageous due to their exceptional encapsulation efficiency, reliable release properties, and distinctive physicochemical characteristics. NO‐releasing polymeric NPs can be fabricated by incorporating or conjugating NO donor molecules into the polymer core, or by modifying the surface of NPs. Due to their nano‐size, NP can readily penetrate target tissues, demonstrating exceptional absorption, distribution, metabolism, and excretion properties.^[^ ^161^ ^]^ Natural, synthetic, semisynthetic, and a combination of natural and synthetic polymers have been used to fabricate desired polymeric NPs for NO cargo delivery. To have a substantial therapeutic effect, the choice of polymers, design, biodegradability, and non‐toxicity are usually key factors taken into consideration while developing polymeric NPs.^[^ ^162^ ^]^ To increase half‐life, biodegradability, and loading efficiency, polymer‐based NPs (e.g., chitosan, dextran, polyethylene glycol (PEG), alginate, and gelatin) have been developed for biomedical applications such as anti‐infection, antioxidant and anti‐inflammatory activities.^[^ ^163^ ^]^ Also, by increasing both the bioavailability of NO and its stability in circulation, polymers, such as PEG‐based delivery platforms (loaded with organic NO donors) improve the release and therapeutic effect of NO.^[^ ^163b^ ^]^ However, one of the concerns in this study is the lack of validation regarding the biocompatibility of these new formulations, which is crucial to ensure the absence of toxic byproducts. Additionally, no specific applications or evidence supporting the effectiveness of these formulations in the mentioned areas of treatment were demonstrated, raising further questions about their efficacy.

To exert a significant therapeutic effect and eliminate the need for frequent dosage administration, NO should have a sustained release from its donor for an extended period. Polymers such as poly (lactic‐co‐glycolic acid) (PLGA)/poly(glycolide‐lactide) (PGLA)‐polyethyleneimine (PEI) based NPs are suitable NO donors due to their enhanced biodistribution which is attributed to their prolonged and sustainable release profiles. Jeong et al. developed NP systems capable of releasing NO, aiming to address the challenges associated with controlling NO release due to its rapid diffusion properties and potential cytotoxicity.^[^ ^164^ ^]^ In their first study, the authors developed an NP system utilizing surface‐modified silica NPs (Si NPs) coated with branched polyethyleneimine (BPEI). This formulation enabled sustained NO release while minimizing initial burst emissions. The resulting BPEI‐coated NO‐releasing Si NPs (BPEI‐NO NPs) exhibited multifunctional properties, including bactericidal efficacy and favorable cell viability for human cells. Additionally, improved ocular wound recovery was observed in a mouse keratitis model. In another study, the authors demonstrated the fabrication of branched polyethyleneimine/alginate (BPEI/ALG) nano‐blended coatings and PLGA nanoparticle‐based BPEI/NONOate for the controlled release of gas molecules.^[^ ^165^ ^]^ By introducing structural heterogeneity to the coating through self‐assembly, the system achieved significant NO release.

The loading efficiency of NO donors into a polymeric system can be modulated by the availability of functional groups, hydrophobicity, biodegradability, and surface charge as well as through the approach of loading and nanoparticle synthesis. Different molecular weights of PLGA were used to study the release of S‐nitroso‐N‐acetylpenicillamine (SNAP) from NPs and the subsequent generation of NO. NPs made of PLGA with a molecular weight of 24–38K exhibited a substantial NO release for ten days. While NPs made of PLGA with a molecular weight of 38–54K showed a sustained NO release over one month. The burst release of NO was observed upon exposure of this delivery system to light irradiation. Hence, the NO emissions can be manipulated to take place over a few hours to a month, depending on the treatment requirements. The PLGA NPs‐based NO donor remained stable at room temperature for a year, suggesting the possibility of easy shipment and long‐term storage capabilities of these conjugates.^[^ ^166^ ^]^

In conjugation with NO donors, PEI can significantly enhance therapeutic outcomes due to its enhanced permeability into the cells. However, quick protonation of the amine groups on the backbone of PEI, or rapid diffusion of PEI, leads to the burst release of NO donors from the conjugates. To resolve this problem, Nurhasni et al. chemically conjugated NO donors with the secondary amine of PEI and then incorporated the conjugates into PLGA NPs. PEI/NO donor‐loaded PLGA NPs exhibited controlled hydrolysis causing a prolonged release of NO for extended periods and significantly enhanced bactericidal activity against MRSA.^[^ ^167^ ^]^ In another study, the NO‐releasing PEI/NONOate‐doped PLGA NPs were synthesized and were able to maintain their antibiofilm activity for a prolonged period. The results illustrated that MRSA biofilms in diabetic mice wounds were substantially destroyed by releasing NO from PEI/NONOate‐doped PLGA NPs. This resulted in the acceleration of wound healing.^[^ ^168^ ^]^ The antibacterial activity of NO primarily depends on the concentration of NO at the site of the microbial biofilm. The available NO released/generated in many pathological conditions is ineffective in completely eradicating bacterial biofilms. But the co‐delivery of drugs/antibiotics/antimicrobial peptides and NO donors for a synergistic effect can be a promising approach. Boyer and coworkers developed a NONOate‐gentamicin complex that co‐delivered NO donors and gentamicin to *P. aeruginosa* biofilms. In this work, they synthesized gentamicin‐conjugated PEGMA NPs, and NO donors were subsequently conjugated to the primary and secondary amines of gentamicin. The combinational therapy synergistically decreased bacterial cell viability and improved biofilm eradication compared to gentamicin or NONOate alone. In addition, the enhanced anti‐tumor effect of NO in combination with chemotherapeutic agents, such as doxorubicin, cisplatin, etc. was observed due to their synergistic effect.^[^ ^169^ ^]^

### 3.2 NO Donating Dendrimers

Dendrimers are a highly branched class of polymers, synthesized in a stepwise manner to contrive a specialized structure. These well‐defined and spherically shaped dendrimers are classified into Generation 1 (G1), Generation 2 (G2), Generation 3 (G3), and so on based on their branching.^[^ ^170^ ^]^ Dendrimers consist of distinct parts, such as the primary core, branch domain, and terminal backbone of functional groups, and can reach up to 100 nm in diameter.^[^ ^171^ ^]^ Their multifaceted functionality, due to their dynamic multivalency, make dendrimers a suitable macromolecule for drug delivery.^[^ ^172^ ^]^ Since regulating the branching of dendrimers based on their synthesis procedure and generation is unfavorable, the surface functionalization of dendrimers along with the desired number of NO groups can be achieved via chemical conjugations. Hence, the dendrimers‐based system provides high NO storage and disseminates substantial amounts of NO, thereby providing evidence of its ability to act as a potential platform for donating NO. In addition to the bio‐mimicking structure, they exhibit excellent polydispersity index (PDI) values, drug‐loading capacity, and controlled release properties.^[^ ^173^ ^]^

In 2008, Stasko et al. reported for the first time the potential of dendrimer‐based scaffolds as NO delivering platforms. They modified G3 and polypropylene imine by employing primary and secondary amine reactions to study the loading efficacy of N‐diazeiumdiolate.^[^ ^170^ ^]^ The authors further examined the effect of functional groups, alkyl chains, and amide reactions on the release of NO from the cargo. They concluded that the conjugation of NONOate and secondary amine dendrimers facilitated NO loading, accounting for 5.6 µmol mg^−1^. The protonation of the donor at a pH of 7.4 led to the release and generation of NO at a maximum level for 16 h. Based on this concept, a series of NONOate‐functionalized polypropylene imine dendrimers were developed to study their bactericidal actions against pathogenic microorganisms, such as *P. aeruginosa*, *E*. *coli*, *S*. *aureus*, and MRSA.^[^ ^170^ ^]^ NO‐releasing dendrimers significantly decreased gram‐positive and gram‐negative bacteria growth compared to non‐NO‐releasing dendrimers and exhibited minimum cytotoxicity to fibroblasts. The bactericidal actions of these NO‐releasing dendrimers are dependent on their molecular weights and functionality. For instance, observation revealed that dendrimers with higher molecular weight generate significantly more NO (8‐10 times) compared to smaller‐sized dendrimers. Similarly, the bactericidal action of styrene oxide (SO) functionalized dendrimers was more effective than PEG functionalized dendrimers.^[^ ^171^ ^]^ The notable bactericidal activity observed in SO‐functionalized dendrimers can be attributed to their high cationic charge and the presence of multivalent benzyl alcohol‐like moieties derived from styrene. These properties disrupt bacterial membrane through enhanced electrostatic interactions and alteration of bacterial protein synthesis. Conversely, in the case of PEG‐modified dendrimers, the cationic charge is reduced due to PEG functionalization, leading to a significant decrease in electrostatic interaction between the dendrimers and bacteria. Consequently, this reduction in electrostatic interaction results in a lower antibacterial activity for PEG‐modified dendrimers. Hence, N‐diazeiumdiolate/polypropylene imine conjugate reported by Stasko et al. could effectively combat severe infections.

In a different study, Liu et al. observed that NO‐donating dendrimers, the surfaces of which were modified with hydrophilic and hydrophobic moieties in equal proportion showed higher efficiency in eradicating biofilms.^[^ ^174^ ^]^ Scientists also attempted to examine the combinatory effect of the co‐delivery of NO and antibiotics in enhancing bactericidal activity. One such study, using the synergistic approach of co‐delivery of methicillin and NO, elucidated a promising treatment approach against gram‐positive, gram‐negative, and MRSA‐induced bacterial infections.^[^ ^174b^ ^]^ The study reported that a NONOate‐functionalized poly(amidoamine) and low molecular weight chitosan conjugation was able to simultaneously deliver methicillin and NO in a controlled release manner, which led to a significant killing effect on bacteria and improved wound healing. Ornelas and co‐workers reported a sustained release of NO by using dendritic structures and subsequently indicated their therapeutic role in inflammatory‐associated pathologies.^[^ ^175^ ^]^ Recently, the same group fabricated a large and dynamic 108 termini‐bearing bifunctional dendrimer for NO and ursodeoxycholic acid (UDCA) release (54 moieties for each). The NO and UDCA‐releasing bifunctional dendritic structure exhibited substantial inhibition of IL‐8 production demonstrating their synergistic anti‐inflammatory activity.^[^ ^176^ ^]^

### 3.3 NO Donating Liposomes

Liposomes represent special spherical vesicles made of natural or synthetic lipids and cholesterols. They are comprised of a hydrophobic backbone and hydrophilic core which enables their efficient loading and delivery of hydrophobic and hydrophilic drugs. Their physicochemical properties vary according to their composition and fabrication techniques. Liposomes are currently used extensively as an NO carrier and delivery system for multiple medical applications.^[^ ^174a^ ^]^ The lipid bilayer structure prevents the rapid decomposition of liposomes; to which the greater stability of the incorporated NO donors and prolonged release kinetics in physiological conditions are attributed. Since liposomes are safe and biodegradable, their application as a NO donor does not pose any related toxic effects. However, the use of liposomes in cutaneous delivery is restricted by their hydrophobic outer layer.^[^ ^163a^ ^]^

Many techniques, such as physical interaction, incubation, or chemical conjugation, have been developed to attach or incorporate NO donors to liposomes. Liu et al. showed a liposome‐based sphingosine‐1‐phosphate (S1P)/JS‐K (NO prodrug) delivery system that was used for the local and targeted delivery of NO from its donor against glioblastoma multiform (GBM), (one of the primary invasive tumors of the central nervous system (CNS)). This system promoted continuous NO delivery via glutathione S‐transferase (GST) overexpression, causing significant GBM cell death.^[^ ^177^ ^]^ The half‐life of NO is relatively short, ranging from a few seconds to several minutes, and can even extend to hours depending on the type of donors used, such as NONOates, which are exogenous and nucleophilic NO donors.^[^ ^178^ ^]^ To prolong the half‐life and enhance the circulation time of NONOates to achieve the desired therapeutic effect, they can be incorporated into the hydrophilic core of liposomes. Further, the bioavailability of NONOates is increased when incorporated into the liposome core, as their outer hydrophobic shell controls the spontaneous release of this water‐soluble NO donor by inhibiting the passage of protons through the phospholipid bilayers. A good example encompasses the encapsulation of spermine diazeniumdiolate into the temperature‐sensitive structure of liposomes.^[^ ^179^ ^]^ Another study showed that the half‐life of NONOate was increased when they were incorporated into a PEGylated liposome.^[^ ^180^ ^]^ Owing to its enhanced permeability and retention effects, an elevated accumulation of NONOate‐loaded PEGylated liposomes was observed in tumor tissues in contrast to normal tissues. The incorporation of NONOate into liposomes can serve as an effective therapeutic strategy for the target‐specific delivery of NO donors, in combination with chemo drugs or immunotherapeutic, to promote anti‐tumor effects. Suchyta et al. reported that NO donor‐incorporated liposomes significantly reduced the viability of mammalian pancreatic cells.^[^ ^181^ ^]^ NO‐donating liposomes play a multifaceted role as they can release NO for extended periods, can be regulated in response to the pH, and are effective against tumors.^[^ ^182^ ^]^

NO‐based bioactive gas delivery properties can also be improved by modulating the successful release of NO using liposomes. Huang et al. developed a unique liposomal system that is echogenic and capable of co‐encapsulating and delivering argon and NO gases simultaneously.^[^ ^183^ ^]^ They showed a 50% spontaneous NO release from echogenic liposomal vesicles (NO‐ELIP) within 10 min of treatment, which was sustained for the next 8 h of the study. In an in vivo study, the local administration of NO‐ELIP effectively inhibited intimal hyperplasia. Lipid‐based systems mainly deliver low molecular weight NO donors either by themselves or in combination with other therapeutic molecules. The fabrication of cholesterol moieties in a lipid‐based system serves to encapsulate NO gas and can aid in the controlled release of NO for diverse biomedical applications. A more effective role of cholesterol linkage in liposomes was later demonstrated by Nakanishi et al. who showed that cholesterol enhances donor affinity to carriers and enables liposome transport via the lipid membrane and generation of NO.^[^ ^184^ ^]^

### 3.4 NO Donating Stimuli Sensitive Materials

Regarding drug delivery, smart materials or stimuli‐responsive materials have attracted considerable attention within the scientific community. The establishment of spatiotemporally controlled delivery systems and NO‐releasing donors in this category is no exception. Stimuli‐responsive materials for NO release have elucidated a promising approach to overcome major limitations associated with conventional delivery systems.^[^ ^185^ ^]^

Gu et al. demonstrated the efficacy of dual responsive self‐assembled NPs in controlled NO release and targeted antitumor effects through in vitro and in vivo studies.^[^ ^185d^ ^]^ The self‐assembly of NPs was achieved by utilizing disulfide and ester linkages in the dimer of phenylsulfonylfuroxan (FZ), which serves as a NO‐generating prodrug. To enhance active tumor targeting, the prodrug dimer (FZ‐SS‐FZ) NPs were surface‐modified with folic acid, resulting in a promising effect. These developed NPs exhibit high NO payload capacity and significant NO release triggered by the presence of high levels of GSH and esterase expression. The NPs demonstrated tumor‐targeting drug delivery, improved drug loading efficiency, and stabilization of NO. However, further improvements are needed for the system to minimize the need for multiple doses and achieve prolonged NO release. In another study, Duan et al. developed amphiphilic vesicles that exhibited a sequential release of NO and gentamicin sulfate.^[^ ^186^ ^]^ The NO release was mediated by the cleavage of N‐NO bonds on exposure to visible light while vesicle disintegration led to the release of gentamicin sulfate. These vesicles could eradicate biofilms and kill dispersed planktonic bacteria.

Another study showcased the utilization of protoporphyrin (PpIX)‐based polymer nanoplatforms, which effectively integrated both photothermal therapy (PTT) and NO delivery. This novel approach raised intracellular temperatures and NO concentrations, resulting in the inhibition of cancer cells both in vitro and in vivo.^[^ ^187^ ^]^ This light‐sensitive multifunctional therapeutic system also helps to resist the onset of multidrug resistance. While the utilization of photo‐responsive approaches has shown promise in facilitating efficient NO release, it is crucial to thoroughly evaluate certain notable concerns associated with this approach. One limitation to consider is the restricted penetration depth of laser‐based PTT in deep tumor tissues.^[^ ^188^ ^]^ This could potentially hinder the effective release of NO if solely dependent on photothermal stimulation, leading to suboptimal NO release and limited therapeutic outcomes, particularly in larger volumes of cancerous tissue. Another critical consideration is the potential induction of heat shock proteins by tumor cells as a response to thermal stimulation during PTT. These proteins serve as a defense mechanism, enabling tumor cells to evade apoptosis and potentially compromising the desired therapeutic applications.^[^ ^189^ ^]^ To achieve effective anticancer effects, NIR‐based strategies have demonstrated success in penetrating deep tumor tissues and should be further explored. In one such study, Chan and coworkers synthesized near‐infrared radiation (NIR)‐light‐responsive NO donors containing an aza‐BODIPY dye appended with an aryl N‐nitrosamine NO‐donating moiety.^[^ ^190^ ^]^ The NO donor molecules (photoNOD‐1 and photoNOD‐2) demonstrated the ability to release NO at the desired site in response to NIR‐light stimulation without requiring multiphoton activation. In the presence of a stimulus, the efficacy of NIR‐photoactivatable photoNOD‐1 in inhibiting tumor growth of murine breast cancer mice was significant. To enhance the potential therapeutic efficacy of stimuli‐responsive materials, alternative approaches in NO‐based combinatorial strategies can be explored. These approaches may involve the use of HSP inhibitors, nanocoating for controlled light conversion, and the employment of suitable in vivo models.^[^ ^188^, ^191^ ^]^ By incorporating HSP inhibitors, the defensive mechanisms of tumor cells can be targeted, potentially sensitizing them to NO‐based therapies. Additionally, utilizing nanocoating that enables precise control of light conversion can enhance the specificity and efficiency of photothermal or photo‐responsive NO release. Furthermore, conducting studies in appropriate in vivo models will provide valuable insights into the effectiveness and feasibility of these advanced combinatorial approaches.

### 3.5 Other Carriers for NO Release

Silica NPs are one of the commonly used NO donors for local delivery of NO. The simple and easy mode of synthesis, tunable surface chemistry and size, sustained release, and non‐toxic properties make silica NPs a potent potential NO delivery carrier.^[^ ^192^ ^]^ Silica NPs as small as 30 nm in diameter were synthesized via amino silane template surfactant ion exchange reaction and demonstrated the capability to enhance the payload of NO inside the NPs and trigger their sustained release.^[^ ^193^ ^]^ NO‐releasing silica NPs can induce a significant bactericidal action that is tunable according to shape, particle size, and surface properties.^[^ ^194^ ^]^ NO‐releasing silica nanospheres with diameters within the range of 14–50 nm were more potent against both gram‐positive and gram‐negative bacteria compared to larger ones.^[^ ^194c^ ^]^ In another study, a higher aspect ratio of silica NPs with smaller sizes was also shown to contribute to anti‐bacterial activities.^[^ ^195^ ^]^ The NO‐releasing characteristics of silica NPs can be tuned via surface modifications. In a similar study, the authors demonstrated the surface modification of silica NPs with PEG, reduced the half‐life of the donors through their decomposition, which occurred due to the large water absorption capacity of PEG, resulting in faster degradation of the delivery vehicle. This type of NPs exhibited enhanced antibacterial properties because of an increase in NO flux in the biological environment.^[^ ^194c^ ^]^ Other than silica NPs, gold (Au) NPs represent another promising option as NO donors.^[^ ^196^ ^]^ The unique physiochemical properties of AuNPs enable efficient transport and release of desired drugs, making them highly versatile for loading and continuous release of NO.^[^ ^178^, ^197^ ^]^ The synthesis of AuNPs is straightforward, and their inert and non‐toxic core allows for safe utilization. Furthermore, AuNPs can be functionalized in a tunable manner, adding to their versatility in drug delivery. They can respond to various internal or external stimuli such as pH, GSH, and light, triggering the release of NO.^[^ ^178^ ^]^ The presence of AuNPs facilitates the formation of S–Au bonds, which have lower dissociation energy compared to S–N bonds, resulting in the release of NO.^[^ ^198^ ^]^ Studies have demonstrated that increasing the concentration of AuNPs enhances NO generation due to the larger surface area available.^[^ ^198a^ ^]^ Surface grafting of AuNPs using block copolymers can achieve slow and controlled NO release, exhibiting significant antibiofilm activity.^[^ ^197a^ ^]^ Additionally, the development of a NIR‐triggered NO nanogenerator (SNP@MOF@Au‐Mal) demonstrated a synergistic antibacterial effect against *P. aeruginosa* infection when exposed to NIR irradiation.^[^ ^185b^ ^]^ Despite the continuous advancements in the development of AuNPs for therapeutic applications, challenges persist that limit their translational potential.

In addition to the existing NPs, a highly interconnected porous structure with a large surface area and tunable physicochemical properties have made metal‐organic frameworks (MOFs) a novel NO donor carrier.^[^ ^199^ ^]^ The properties of MOFs such as metal‐center or organic linkers can be tuned according to the demand. The tunable pore size facilitates the loading of NO to MOFs. In addition, chemical conjugation is another possible way to incorporate NO into MOFs. Several studies demonstrated that MOFs could accomplish the controlled delivery of NO at a target site. For example, one of the studies reported the prolonged release of NO for 7 days using Cu‐TDPAT‐type MOFs.^[^ ^200^ ^]^ However, the biocompatibility and biodegradability of MOFs are still under question. To address these issues, Bloch et al. developed iron‐II‐based MOFs which are dependent on the biocompatible profile of iron. They demonstrated that the Fe_2_(dobdc) MOFs facilitated a burst release of NO within the first 72 h; for the next 96 h, Fe_2_(dobdc) exhibited slow‐release properties. The sustained release of NO was further improved to last for at least 10 days, which was ascribed to the strong binding of NO to the Fe‐based MOF.^[^ ^200^, ^201^ ^]^ However, these MOFs only released NO by reacting with or displacing water molecules. Subsequently, one study showed that the developed MOFs usually do not release NO under normal conditions; however, upon light activation, they can release a significant amount of NO from the MOFs.^[^ ^202^ ^]^ This study illustrated that photo‐responsive donors can release NO in a controlled manner in the cellular environment upon exposure to laser radiation.

However, the utilization of NO donors in conjunction with metal‐organic frameworks (MOFs) does present certain drawbacks. A significant concern revolves around the potential toxicity of MOFs when exposed to physiological conditions. Additionally, there is a need for comprehensive validation of stimuli‐responsive parameters, taking into account factors such as intensity, wavelength, and duration, and their impact on essential physiological parameters. Neglecting to address these factors could raise apprehensions regarding the controlled and safe administration of NO using MOFs. Furthermore, to fully evaluate the feasibility and safety of employing NO donors with MOFs, it is imperative for future research to thoroughly investigate and discuss the concentration and biocompatibility of MOFs under physiological conditions.

## 4 Biomedical Applications

The multifaceted roles of NO in the biological framework have amassed great interest in the development of strategies to deliver exogenous NO for biomedical applications. The use of systems for NO delivery is instrumental in the strategies of accomplishing controlled and sustained release of NO to different tissues and organs. NO‐delivery systems have shown promising results in numerous biomedical applications such as wound healing, cardiovascular homeostasis, ischemic therapy, and treatment of infections and several types of cancer. The following section summarizes the progress of various NO‐delivery systems utilized in the biomedical field.

### 4.1 Wound Healing and Skin Repair

Wound healing is a natural process and progresses in regulated and sequential phases involving hemostasis, inflammation, proliferation, and remodeling which requires the involvement of numerous growth factors, cytokines, and cellular elements.^[^ ^203^ ^]^ NO plays several roles in wound healing. NO has been observed to stimulate the growth and proliferation of fibroblasts, keratinocytes, and endothelial cells.^[^ ^173^, ^204^ ^]^ The sustained presence of NO during wound healing has been demonstrated to increase fibroblast migration and collagen deposition in granulation tissue.^[^ ^205^ ^]^ NO also contributes to improved wound healing by upregulating angiogenic factors, such as TGF‐ß and VEGF, which ensures adequate blood supply for healing. However, in cases of impaired wound healing, inadequate NO synthases and low levels of available NO lead to decreased collagen deposition, unregulated inflammatory responses, tissue hypoxia, and prolonged healing time.^[^ ^206^ ^]^ Therefore, a balanced level of NO is a pre‐requisite and can be maintained by supplementing it through endogenous and exogenous pathways.

**Figure 3:**
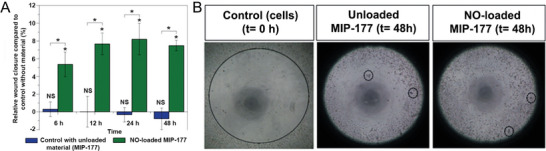
The role of NO in cell migration and wound closure. A) MIP‐177 and NO loaded MIP‐177 effect on wound closure in in‐vitro for 48 h. B) Microscopic images showing the pre‐migration stage without detection mask at time = 0 h and the migration of cells detected with a mask after 48 h. The black circles indicate the presence of MIP‐177 particles between the migrated cells. Reproduced with permission.^[^ ^207b^ ^]^ Copyright 2020, John Wiley and Sons Inc.

Many exogenous NO donors have been stabilized for longer NO release profiles and have shown promising efficacy in wound healing.^[^ ^206^, ^207^ ^]^ In the case of non‐obese, diabetic, and/or severe combined immunodeficiency (NOD‐SCID), wound healing has always been an utmost point of research interest. Controlled NO release is important in dealing with such wounds because a prolonged period of treatment is needed for effective wound closure. Blecher et al. synthesized NO‐enriched NPs (NO‐NPs) made of hydrogel‐glass composite containing a mixture of tetramethylorthosilicate, polyethylene glycol, glucose, chitosan, and sodium nitrite.^[^ ^206a^ ^]^ They tested the release profiles of NO from the NO‐NPs in wounds created in eNOS and iNOS knock‐out NOD‐SCID mice. The NO‐NPs showed release rates of NO with ≈70 nm min^−1^ in the first 10 min, ≈180 nm min^−1^, and ≈215 nm min^−1^ in the next 100 and 200 min, respectively. In addition, the NO‐NPs showed a NO release rate of ≈245 nm min^−1^ from 5 to 24 h. The group treated with NO‐NPs achieved 29.4% and 84% wound closure on day 5 and day 9 respectively. Whereas the control group showed 12.5% and 55.5% wound closure within the same periods. This study has shown a great prospect for the sustained delivery of exogenous NO in the treatment of wounds in mice with severe immune dysfunction (e.g., lack of CD4^+^ and CD8^+^ T lymphocytes causing severely impaired fibroblast replication and collagen synthesis). The sustained NO delivery mainly promoted fibroblast migration, collagen deposition, and enhanced angiogenesis which is significant during the proliferative phase of wound healing. Pinto et al. showed the significance of controlled NO release in tuning cellular functions by using titanium‐derived metal‐organic frameworks (Ti‐MOF) as reservoirs of NO, which were named MIP‐177.^[^ ^207b^ ^]^ The study showed the potential of these porous NO donating structures as an ideal candidate for skin reparatory therapeutic agents. The MIP‐177 showed a maximum NO storage capacity of 9% w/w which was achieved mainly through in situ adsorption. The use of NO‐loaded MIP‐177 resulted in the transformation of oxyhemoglobin into methemoglobin, which was attributed to the release of NO. The wound closure was mainly tested in vitro by examining the migration of HeLa cells and mitochondrial respiration via measurement of the oxygen consumption by the cells (**Figure** **3**). The migration of HeLa cells showed 8% wound closure after being treated with 90 µm mL^−1^ NO‐loaded MIP‐177 while regular MIP‐177 treated cells showed marginal wound closure in 48 h (Figure 3A). Figure 3B shows phase contrast images which indicate that a significantly higher number of cells migrated into the defect of the NO‐loaded MIP‐177 treatment group when compared with the MIP‐177 group.

**Figure 4:**
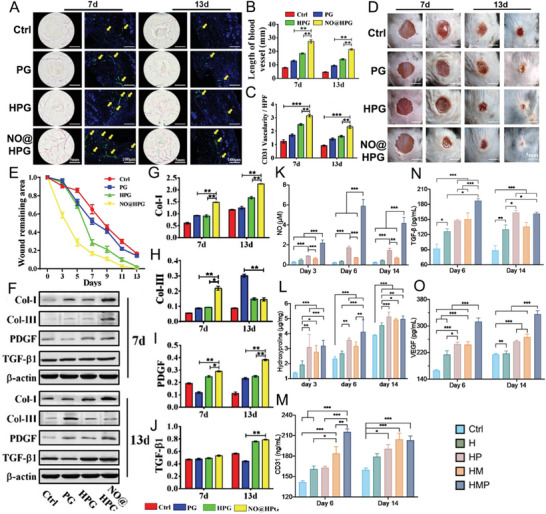
Effect of NO‐releasing scaffold on angiogenesis and wound healing. A) Representative images showing neovascularization and respective CD31 expression (blood vessels stained in green). B) Presents quantitative data of the length of vessels and C) CD31 stained cells, respectively. D) Digital images showing the effect on wound healing by different groups and E) Corresponds to wound remaining area at preset time points. F–J) Gene expression of in vivo wound tissue samples on days 7 and 13 by western blotting analysis. Adapted with permission.^[^ ^208^ ^]^ Copyright 2020, American Chemical Society. Quantitative analysis of growth factors in new skin tissues of wound K) NO, a repair factor, L) Hydroxyproline, for collagen deposition, M) CD31, N) TGF‐β, and O) VEGF. Reproduced with permission.^[^ ^212^ ^]^ Copyright 2022, Elsevier Ltd.

In another work, Zhang et al. loaded NO‐containing Cu‐MOF NPs into PCL‐Gel composite scaffolds (NO@HGP) and examined endothelial cell viability, proliferation, migration, and healing properties of NO@HGP in diabetic wounds.^[^ ^208^ ^]^ The study showed a prolonged release of NO from NO@HGP over the course of 10 days and revealed the formation of more capillary networks in the NO@HGP group when compared to control groups. NO also promoted the expression of angiogenic factors, leading to neovascularization needed for an adequate supply of blood to the wound (**Figure** **4**A‐C). As shown in Figure 4D, the digital images displayed efficient healing in the NO@HGP group in contrast to the other control groups. Only around ≈15% of the wound remained open after 7 days of treatment (Figure 4E). Further, the study showed an increase in re‐epithelization as well as the formation of blood vessels in NO‐treated groups when compared to other groups as verified by the high expression of Col I, PGDF, and TGF‐ß in the NO@HGP treated wounds (Figure 4F‐J). The expression of Col I, PGDF, and TGF‐ß was believed to contribute to granulation tissue formation and tissue remodeling, by increasing the expression of genes associated with the formation of the ECM, including protease inhibitors and collagen.^[^ ^209^ ^]^

The major role of a sustained release of NO in the case of wound healing is related to its role in tuning angiogenesis. Rapid angiogenesis in the region of the wound is mostly advantageous for fast healing because the rapid formation of vascular structures leads to an enhanced oxygen supply, rapid collagen deposition, and epithelization.^[^ ^206^, ^210^ ^]^ The angiogenic growth affects the proliferation and association of the endothelial cells that form lumen‐like structures for blood vessels in the region of the wound.^[^ ^211^ ^]^ NO‐generating multifunctional HMP hydrogel was fabricated by Tu et al. to promote accelerated healing in an infected diabetic wound by exploiting its ROS scavenging activity, anti‐inflammatory role as well as ability to promote angiogenesis.^[^ ^212^ ^]^ The HMP hydrogel, containing a NO synthesizing enzyme (pravastatin sodium), showed elevated NO generation through eNOS activation and the amplification of TGF‐β, VEGF, and total collagen content (Figure 4K‐O). The study further elucidates NO's role in reducing the inflammatory response, inducing vascular permeability, and increasing neovascularization thereby promoting rapid wound healing. Zhang et al. incorporated sodium nitrite into gelatin‐siloxane NPs (GS‐NO NPs) for NO delivery. The effect of the sustained release of NO from the GS‐NO NPs was observed through the cellular proliferation of human umbilical vascular endothelial cells (HUVECs), which is a standard model for in vitro endothelial proliferation studies. GS‐NO NPs at the concentration of 100 µg mL^−1^ showed a 41.7% increase in the HUVECs when compared to an untreated negative control which was just 27.7% on day 7. The cell viability was also observed to be 190% of the negative control. An increased association of the HUVECs forming thicker wall‐like structures was observed. NO at low concentrations (10 nm to 1 mm) promotes the proliferation of HUVECs, induced by VEGF and bFGF expression and subsequent activation of the MAPK pathway.^[^ ^211^, ^213^ ^]^

In skin regeneration, apart from the well‐established role of NO in cell growth and migration, angiogenesis, matrix deposition, and re‐epithelization, NO exhibits a pivotal role in reducing inflammation and the remodeling of injured tissue. NO can modulate inflammatory signals (e.g., TNF‐α) and immune cells (e.g., dendritic cells) to induce a substantial anti‐inflammatory response.^[^ ^214^ ^]^ Wan et al. fabricated a polyurethane (PU)/Gel composite containing a keratin (K) based NO donor (SNO) and demonstrated its potential application in wound repair.^[^ ^215^ ^]^ The study illustrated that in contrast to a control group, NO‐releasing PU/Gel/KSNO mats can decrease TNF‐α levels thereby lowering pro‐inflammatory activity. In addition, the presence of improved vascularization, as well as increased collagen deposition and thickness of granulation tissue in the PU/Gel/KSNO group indicated that NO has a significant role in accelerating skin tissue repair and regeneration. In addition, one of the markers of proper skin regeneration is generally considered to be scarless healing. This remains one of the major concerns associated with the healing of deep injury wounds and demands advanced and improved methodology for scarless healing. Recently, NO‐releasing sponges/hydrogels have displayed the ability to significantly reduce scar formation in burned/diabetic wounds and endow a therapeutic approach that may potentially result in scarless skin repair.^[^ ^212^, ^216^ ^]^

### 4.2 Antibacterial Applications

The free radical NO is a potent antimicrobial agent. Exogenous NO donors almost have a similar effect as endogenous iNOS which helps in the production of large amounts of NO for a longer period providing immune responses against pathogens.^[^ ^115^, ^217^ ^]^ The amount of NO generated (around 360 nm) from RSNO at physiological concentrations is not enough to exhibit antibacterial activity against bacteria like *E. coli* and *S. aureus*.^[^ ^218^ ^]^ Further, wounds associated with pathological conditions (e.g., diabetes and immunodeficiency) are more prone to severe microbial infections, and a higher concentration of NO from exogenous sources is needed to exert a bactericidal effect.^[^ ^218^ ^]^ NO is a lipophilic molecule and can cross the bacterial cell membrane quite easily.^[^ ^219^ ^]^ High intracellular levels of NO are the primary cause of nitrosative and oxidative stress. NO oxidized to N_2_O_3_ creates nitrosative stress within and near the bacterial membrane. On the other hand, peroxynitrite (ONOO‐) which is a product of NO reacting with superoxide (endogenously derived from bacterial cellular respiration) produces oxidative stress.^[^ ^219^, ^220^ ^]^ A successful bactericidal effect in wound regions can be quickly achieved through a prolonged involvement of NO, readily available through exogenous NO donors. Both gram‐positive and gram‐negative bacteria, including MRSA, are susceptible to NO.^[^ ^221^ ^]^

Whenever any wound site is treated by using scaffold‐based tissue regeneration methods, it is always necessary that the scaffold mimics the ECM which supports the regenerating tissue. Another objective that needs to be kept in consideration is the protection of the regenerating wound region from microbial infections. Ghalei et al. showed that NO the standard biomolecule was used for targeted antimicrobial effects. The NO was incorporated into polylactic acid (PLA)‐Honey‐S‐Nitroso‐Nacetyl‐penicillamine (PLA/HN/SNAP) electrospun scaffolds which were also used as the ECM mimicking structure.^[^ ^222^ ^]^ The covalent conjugation of SNAP with PLA using different concentrations of honey enhanced the release profile and prolonged the sustained release of NO from SNAP over a period of 48 h. The anti‐microbial activity of the PLA/HN/SNAP electrospun scaffolds tested against *E. coli* and *S. aureus* revealed a significant reduction in bacterial viability by 94.4% and 96.4%, respectively. The sustained release of NO acted as a potent antimicrobial agent and revealed enhanced cell viability in the PLA/HN/SNAP nanofiber‐treated group, demonstrating that PLA/SNAP‐based scaffolds could be an ideal candidates for wound regeneration. NO donors containing NPs for extended NO release have been of key interest due to their localized delivery efficacy. The larger surface area provided by NPs in the infected site makes them suitable for better in‐contact delivery in wound regions that are prone to bacterial infections. Hetrick et al. synthesized NO‐releasing silica NPs and 1‐[2‐(Carboxylate) pyrrolidine‐1‐ium‐1,2‐diolate (PROLI/NO) for studying the effect of sustained release of NO on *P. aeruginosa*.^[^ ^219b^ ^]^ It was observed that NO delivered from silica NPs could effectively kill *P. aeruginosa* better than NO released from small molecule PROLI/NO, which was attributed to the slower NO release from silica NPs (≈3.5 µmol mg^−1^) than from PROLI/NO (≈21 µmol mg^−1^) after 4 h. The burst release of NO from PROLI/NO also turned out to be more toxic towards L929 mouse fibroblasts than was the gradual release of NO from NO‐releasing silica NPs. The burst release of NO saturates the intracellular spaces causing extensive cytotoxicity in structural cells; while a sustained and moderate release of NO is essential to stop microbial growth. Martinez et al. also exploited the potential of the gradual release of NO from glass/hydrogel‐based composites (NO‐NPs) made out of tetramethylorthosilicate, polyethylene glycol, chitosan, glucose, and sodium nitrite.^[^ ^223^ ^]^ Wounded skin lesions of BALB/C mice were infected with 10^7^ CFU mL^−1^ of MRSA and treated with NO‐NPs, resulting in a significant decrease in the size of the eschar when compared to the untreated group. The bacterial samples collected from the infected and uninfected skin lesions demonstrate a significant decrease in bacterial infection in the group treated with NO‐NP compared to the untreated and NP‐treated groups. Tissue sections from the NO‐treated skin samples showed less inflammation with increased fibrin deposition and no evidence of MRSA.

Not only polymeric scaffolds or NPs are used for conjugating NO donors, but various hybrid or combinatory systems are also used for exogenous NO delivery in intracellular spaces. The attachment of S‐nitroso compounds to various delivery vehicles and targeting the endogenous S‐nitrosothiols in human blood to produce NO are well‐known strategies in the field of antimicrobial studies. Cardozo et al. synthesized S‐nitroso‐mercapto succinic acid alginate/chitosan (S‐nitroso‐MSA alginate/chitosan) and S‐nitroso‐MSA‐chitosan/sodium tripolyphosphate NPs for the treatment of *S. aureus* and *E. coli* and elucidated the prolonged release of NO and its prominent antibacterial activity.^[^ ^217^ ^]^ The main mechanism underlying the antimicrobial activity was the conversion of RSNOs to NO, which induces DNA damage through multiple pathways. NO can directly interact with the DNA structure of bacteria, inhibiting DNA repair mechanisms and impeding the generation of genotoxic products, thereby exerting its antimicrobial effects.^[^ ^115^, ^217^ ^]^ The production of NO from RSNOs can also be catalyzed by the presence of metallic ions such as Cu^+^ which acts as a catalyst to produce 2NO and RS‐SR from 2RSNOs.^[^ ^224^ ^]^ This reactionproduces enough endogenous and exogenous NO for antibacterial activity. Darder et al. used copper‐containing metal‐organic conjugates as the source of copper, delivered through cellulose‐based sponges.^[^ ^218^ ^]^ Copper high aspect ratio structures (CuHARS) were crosslinked with cellulose nanofiber sponges for the release of endogenous NO from RSNOs under simulated conditions in vitro for anti‐bacterial activity as well as in surgical treatment regions. CuHARS releases Cu^2+^ which catalyzes the decomposition of RSNOs to NO over a period of several hours and shows promising inhibition of *S. aureus* and *E. coli* colony formation.

Moreover, *P. aeruginosa* has always been a major cause of nosocomial infections and has been very common in the intensive healthcare units of hospitals. Nguyen et al. created a hybrid antibiotic exogenous NO donating system to control infections related to *P. aeruginosa*.^[^ ^225^ ^]^ NONOate conjugated with 36 repeating units of monomer oligo(ethylene glycol) methyl‐ether‐methacrylate with 3‐vinyl benzaldehyde block polymer (POEGMA‐b‐PVBA) were conjugated to gentamicin to form gentamicin‐conjugated POEGMA‐b‐PVBA NPs with NO (GEN‐NO NP). The combined release of gentamicin and NO was observed over a period of 17 h using a concentration of 5 mg mL^−1^ at pH 7.4, 37˚C. In the presence of GEN‐NO NPs with various concentrations of the NO donor (5–50 µm), the biofilm viability of *P. aeruginosa* was significantly reduced with less than 5% viability in the case of the 50 µm concentration when compared to the untreated cultures which had more than 80% viability after 6 h. The combined therapy of antibiotics with exogenous NO donors has great potential in preventing biofilm formation on clinical surfaces or implants.

**Figure 5:**
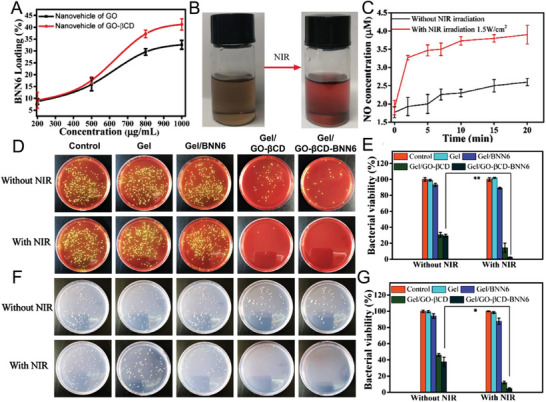
Photothermal effect and NO‐based antibacterial activity. A) Loading ratio (%) of BNN6 (NO donor) in GO‐based vehicles. B) NIR‐based color change of GO‐βCD in the absence and presence of BNN6. C) Release of NO with or without NIR irradiations. D‐G) Agar plates show the antibacterial activity of GO‐βCD‐BNN6 and other groups along with their respective quantitative bacterial viability against *S. aureus* (D,E) and *E. coli* (F,G), respectively. Reproduced with permission.^[^ ^226^ ^]^ Copyright 2020, American Chemical Society.

**Figure 6:**
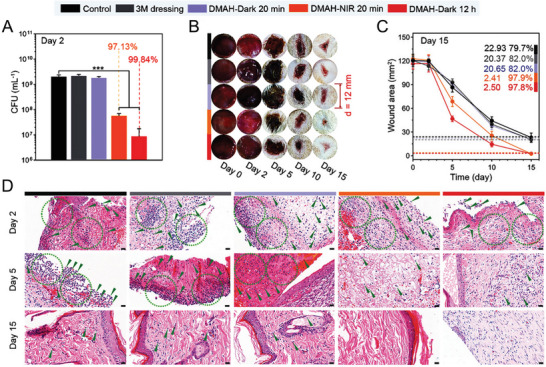
In‐vivo antibacterial and wound healing efficacy of DMAH. A) Antibacterial activity against MRSA on day 2. B) Digital images display wound healing at desired time points after treatment with respective groups and C) Presents the quantification of wound closure on days 2, 5, 10, and 15. D) H&E staining of skin tissue from the wound showing wound healing and neutrophil infiltration (green circles and arrows). All Scale bars are 20 µm. Adapted with permission.^[^ ^227^ ^]^ Copyright 2022, John Wiley and Sons Inc.

To improve the healing of infected wounds, a combined approach was employed by Huang et al. which included NO‐containing graphene oxide (GO) nanocarriers and GelMA/hyaluronic acid graft dopamine‐based photo‐responsive hydrogel.^[^ ^226^ ^]^ The loading of NO donor BNN6 was observed to be higher in the GO‐βCD nanovehicle, and the GO‐βCD‐BNN6 vehicle showed color change due to NO release when irradiated with a NIR laser (**Figure** **5**A,B). On exposure to NIR irradiation, the NO‐GO nanovehicle‐embedded hydrogel exhibited slower and sustained release of NO in comparison to the group without NIR exposure (Figure 5C). The synergistic effect of the developed NO‐containing system demonstrated a potent bactericidal effect against both *S. aureus* and *E. coli* in the presence of NIR irradiation. The NIR and NO‐GO‐based antibacterial activity in *S. aureus* and *E. coli* was found to be promising, as no bacterial colonies were observed, which is clear from digital images of the agar plates. Inhibitory activity against *S. aureus* and *E. coli* was estimated to be ≈97.6% and ≈95.5% respectively, with the highest effect occurring in the Gel/GO‐βCD‐BNN6 + NIR group when compared to the other groups (Figure 5D‐G). Another recent study reported antibacterial GO‐doped dual‐mode hydrogel, incorporated with biomimetic bacteriophage‐like particles.^[^ ^227^ ^]^ The biomimetic phage microparticles were synthesized from *Lactobacillus casei* and combined with RSNOs which served as the NO donor. The study revealed excellent antibacterial activity (≈99.84%) in the dual‐mode antibacterial hydrogel (DMAH) group as compared to the other groups in the study (**Figure** **6**A). The DMAH group was able to effectively eradicate MRSA, and its anti‐inflammatory properties promoted rapid healing in infected wounds with a wound closure of ≈97.8% after day 15 (Figure 6B‐D).^[^ ^212^ ^]^

**Figure 7:**
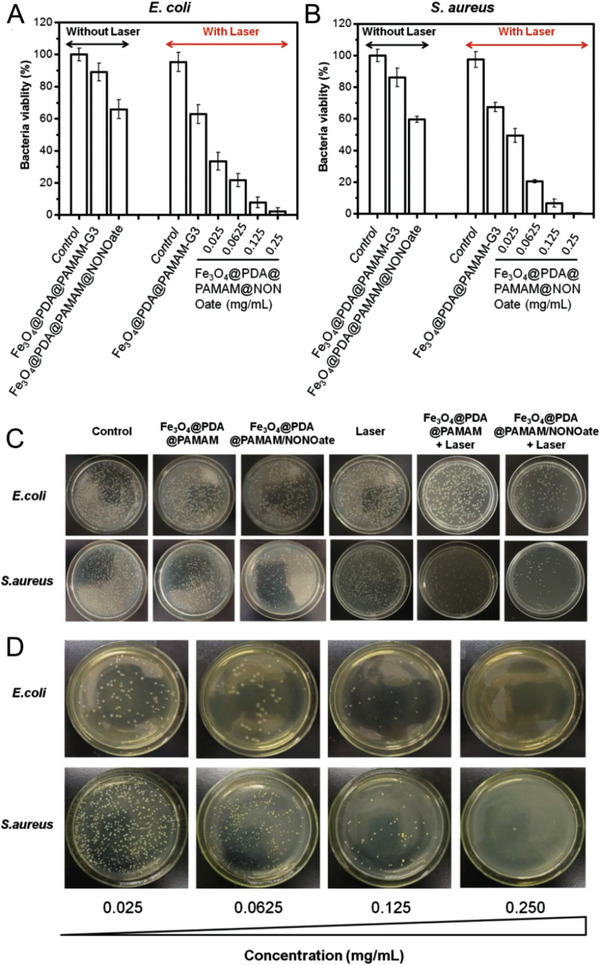
Concentration‐dependent antimicrobial activity of NO under laser irradiation. A,B) Bacterial viability of *E. coli* and *S. aureus* respectively, treated with Fe_3_O_4_@PDA@PAMAM‐G3 and Fe_3_O_4_@PDA@PAMAM@NONOate under different laser irradiation conditions. C) Bacterial colonies formation of *E. coli* and *S. aureus* under different treatments. D) Bacterial colonies formation of *E. coli* and *S. aureus* treated with different concentrations of Fe_3_O_4_@PDA@PAMAM@NONOate. Reproduced with permission.^[^ ^228b^ ^]^ Copyright 2018, John Wiley and Sons Inc.

NIR irradiation and photoconversion of dopamine to polydopamine have been one of the recent advancements in biofilm eradication, but the efficacy of the antimicrobial effect is still a challenge.^[^ ^228^ ^]^ Lei et al. demonstrated a remarkable bactericidal effect of the developed nanocoating based on polydopamine (PDA) under NIR irradiation.^[^ ^229^ ^]^ The bactericidal effect of the PDA‐based nanocoating was found to be independent of the bacterial genus, indicating its broad‐spectrum antimicrobial activity. N‐diaziniumdiolate (NONOate) conjugated with various polymeric NPs has been an effective source for the release of NO which provides bactericidal properties effective against both gram‐positive and gram‐negative bacteria like *S. aureus* and *E. coli*.^[^ ^159^ ^]^ Yu et al. prepared PDA‐coated Fe_2_O_3_ NPs and conjugated them with poly‐(amidoamine) and NONOate as an integrated photothermal nanocomposite and studied the effect of exogenous NO released on populations of *E. coli* and *S. aureus*.^[^ ^228b^ ^]^ The synergistic effect of laser irradiation on the Fe_2_O_3_@PDA@PANAM‐NONOate was studied along with the effect of increasing the dosage of the Fe_2_O_3_@PDA@PANAM‐NONOate (**Figure** **7**). The study highlighted a NO dose‐dependent bactericidal activity in both *E. coli* and *S. aureus*, with exposure to laser irradiation compared to those without laser exposure (Figure 7A,B). The antibacterial effect was determined by counting the colonies using agar plates. All the groups without laser irradiation showed the presence of bacterial colonies; albeit the number of colonies was lesser in the groups treated with Fe_2_O_3_@PDA@PANAM‐NONOate NPs alone when compared to the control group, which demonstrates a weak anti‐bacterial effect (Figure 7C). However, significant antibacterial activity against both *E. coli* and *S. aureus* was observed in the groups when treated with both NO and laser irradiation. It was also ascertained that bactericidal activity increased with increased dosages of Fe_2_O_3_@PDA@PANAM‐NONOate in the presence of laser irradiation (Figure 7D). The bactericidal effect was attributed to the heat generated by the laser irradiation, exposure to which had a significant effect on the destruction of the bacterial cell walls. Another observed advantage of these exogenous NO‐carrying polymeric NPs was the presence of a larger surface area which facilitated interactions with the bacteria colonies. They were also able to reach the colonies more quickly and attach to the bacterial cell walls. Most of the NO donors which served as the exogenous sources of NO have proven themselves to be better delivery vehicles for targeted NO release when compared to the direct use of NO donors (RSNO or NONOate). However, the study did not provide any experimental data to evaluate the effect of NO donor concentrations on bactericidal activity in the absence of laser irradiation, which is a limitation of the study.

### 4.3 Cardiovascular Homeostasis and Inhibition of Platelet Aggregation

NO mediates multiple physiological and pathophysiological processes in the cardiovascular system through various mechanisms such as antiplatelet activity, vasodilation, antioxidant activity, anti‐adhesion, and antiproliferative effects.^[^ ^63^, ^230^ ^]^ Endothelial dysfunction and impaired endogenous platelet inhibition are both part of the cardiovascular phenotype of congestive heart failure (CHF) and usually contribute to increased risk for thromboembolic complications which lead to platelet activation.^[^ ^231^ ^]^ Further, reduced NO activity along with increased levels of ROS results in impaired systemic and coronary perfusion, which needs to be compensated through exogenous boosting of NO‐releasing pathways.^[^ ^232^ ^]^

**Figure 8:**
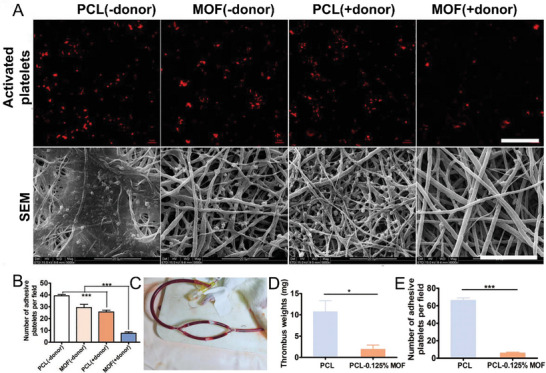
Effect of NO‐releasing scaffolds on platelet adhesion and activation. A) Activated platelets in red (CD62p stain) and corresponding SEM images in PCL/PCL‐MOFs‐GSNO. B) Quantitative estimation of platelet adhesion. C) Digital image presenting ex‐vivo AV shunt model. D,E) Thrombus formation (mg) and adhered platelet count in PCL/PCL‐MOFs‐GSNO scaffolds, respectively. Scale bar: 20 µm. Reproduced with permission.^[^ ^234^ ^]^ Copyright 2021, Elsevier Inc.

Various types of NO donors, like glyceryl trinitrate (GTN), sodium nitroprusside (SNP), diethylamine diazeniumdiolate (DEA/NO), and RSNOs, RIG200 are currently being explored as possible mechanisms to achieve a sustained release of NO in cardiovascular systems.^[^ ^233^ ^]^ Flierl et al. demonstrated low adhesion of activated platelets to fibrinogen in the presence of pentaerythritol tetranitrate (PETN; NO donor) by 50% compared to the control (untreated fibrinogen samples).^[^ ^231^ ^]^ PETN was shown to minimize thromboembolic complications in CHF by interacting with platelet NADPH oxidases and platelet mitochondrial redox reactions to lower the amount of ROS. In recent years, MOFs such as Cu‐MOFs have been elucidated as another excellent source of catalyzing RSNOs endogenously to produce NO for cardiovascular applications.^[^ ^234^ ^]^ By slowing down the rate of spontaneous NO exposure and catalysis, a sustained and extended rate of NO release can be achieved. Zhang et al. embedded Cu‐MOFs in PCL fibers using electrospinning to stabilize MOFs, allowing for a slow rate of NO catalysis, and thereby enhancing NO release for a prolonged period. The sustained NO release prevented platelet adhesion and inhibited platelet activation in PCL/Cu‐MOFs with NO donors compared to the groups without NO donors (**Figure** **8**A,B). The insight into the platelet inhibitory role of NO was further validated in a rat model that used an ex‐vivo arteriovenous (AV) shunt and demonstrated a decrease in clot formation as well as reduced adhesion of platelets in NO‐producing groups (Figure 8C‐E), respectively. However, endogenous NO scavengers like ferro hemoglobin (Fe(II)Hb) and selective guanylate cyclase inhibitor, H‐(1,2,4) oxadiazole (4,3‐a) quinoxaline‐1‐one (ODQ) are also present which may interfere with sGC activation, thereby affecting proper NO binding. So, the effect of different NO donors in the presence of these scavengers needs to be properly exploited to better understand the aggregation effect of the activated platelets.^[^ ^233a^ ^]^ Sogo et al. reported not only the platelet inhibitory effect of each NO donor (e.g., GTN, SNP, GSNO, DEA/NO, and RIG200) but also the effect of endogenous NO scavengers on the attachment of activated platelets to a collagen treated surface. Fe(II)Hb and ODQ inhibited the effect of the NO donors. With increasing concentrations of the NO donors (e.g., SNP, GSNO, RIG200, and DEA/NO), the platelet aggregation decreased rapidly even in the presence of the NO scavengers.

NO, a well‐known vasodilator, is endogenously synthesized via endothelial NO synthase (eNOS) and induces smooth muscle relaxation to expand arteries, which serves a critical role in cardiovascular homeostasis and inflammatory responses.^[^ ^190^, ^235^ ^]^ Various pathological conditions such as diabetes, coronary artery disease (CAD) and CHF usually disrupt normal cardiovascular homeostasis, leading to an increased mortality risk in patients with these conditions.^[^ ^236^ ^]^ Vascular grafts, stents, or other cardiovascular implants containing exogenous NO donors are currently being developed to fully harness the therapeutic potential of NO's effects on the cardiovascular system.^[^ ^237^ ^]^

Cardiovascular implants, such as vascular grafts, valve replacements, and stents constitute most foreign implants that are responsible for the activation of platelets and immune‐related responses. The subsequent aggregation of platelets in the region of an implant can lead to severe cardiovascular conditions like angina pectoris, coronary artery disease, and myocardial infarction.^[^ ^230^, ^238^ ^]^ Moreover, one of the major implications in platelet aggregation is the restenosis of a stented segment of an artery after the completion of angioplasty. The restenosis of the stented segment occurs primarily due to the excessive aggregation of platelets on the surfaces of the stent.^[^ ^238^, ^239^ ^]^ One of the ways to address the problems caused by platelet aggregation is to control the behavior of platelet movement. Most of the previously mentioned implant materials are often coated with NO donors, NO released from these donors reduces the tendency of platelets to aggregate when exposed to the implants. Both hydrophilic (e.g., PEG and hyaluronic acid (HA)) and hydrophobic (e.g., PCL, PLGA, and PU) materials, along with NO donors are used to coat stents.^[^ ^230^, ^240^ ^]^ Acharya et al. coated steel stent surfaces with PEG, PCL, and PLGA containing GSNO to validate the NO release profiles of these polymeric coatings, and the effect of NO on platelet adhesion. The study showed that GSNO can act as a regulatory agent for maintaining blood flow, blood pressure, and thrombus formation in a stented artery.^[^ ^240^ ^]^

**Figure 9:**
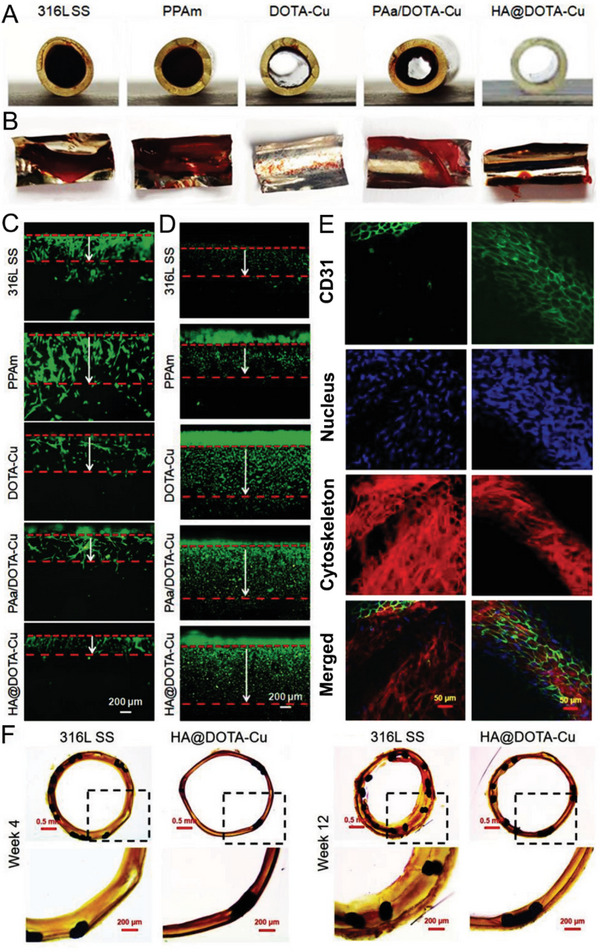
NO containing stent‐cell interaction and in‐vivo implantation. A,B) Digital images of the vascular stent in cross‐sectional view after exposure to blood flow for 2 h and thrombus formation on corresponding foils, respectively. C,D) Effect of developed HA@DOTA‐Cu and other vascular grafts on smooth muscle cell migration and HUVECs, respectively. E) Fluorescent images show cell integration on vascular stent of 316L SS (control) and HA@DOTA‐Cu after implantation. F) Histomorphometric analysis of implanted stents in‐vivo. Reproduced under Creative Commons Attribution License (CC BY).^[^ ^241^ ^]^ Copyright 2021, The Authors. Published by Wiley‐VCH.

To address the problems of thrombosis and restenosis, Lyu et al. fabricated a dual functional endothelial mimicking cardiovascular stent. The plasma polymeric allylamine (PPAm) stents coated with HA and NO generating Cu^2+^‐ 1,4,7,10‐tetraazacyclododecane‐*N*, *N*′, *N*″, *N*″′‐tetraacetic acid (DOTA) in a layer‐by‐layer fashion were evaluated for thrombosis, NO release, cell‐vessel properties in‐vitro and in‐vivo.^[^ ^241^ ^]^ The PPAm stent coated with HA and Cu^2+^‐DOTA not only protected the stent surface from platelet adhesion but also significantly reduced thrombus formation and improved blood flow (**Figure** **9**A,B). Proper endothelialization is a prerequisite at the site of stent surfaces and vascular lesions. Glycocalyx agents and NO are known to be the key contributors to this process. The inhibition of smooth muscle cell growth on stent surfaces is desirable and helps to decrease the chances of restenosis, while higher endothelial cell growth and migration are desired for appropriate endothelialization. The HA scaffolds containing glycocalyx with NO‐releasing properties showed substantial inhibition of smooth muscle cells as well as enhanced endothelial cell adhesion and migration, specifically in HA@DOTA‐Cu (Figures 9C,D). The dual functional HA@DOTA‐Cu vascular stent in‐vivo exhibited excellent migration and integration of endothelial cells within one week (Figure 9E) and demonstrated minimal neointimal hyperplasia after 4 and 12 weeks which is predominantly associated with smooth muscle cell proliferation and migration (Figure 9F). In addition, smaller diameter prosthetics used in bypass surgeries, like vascular grafts, are prone to platelet aggregation and thrombus formation, which is a significant cause of reduced blood flow through and the eventual clogging of prosthetic grafts.^[^ ^242^ ^]^ Fleser et al. also employed a common strategy with the incorporation of NO donors into graft materials for controlling platelet aggregation and thrombus formation in the local regions of the prosthetic application. The polyvinyl chloride (PVC) coated grafts containing NO showed negligible thrombus formation when compared to the PVC‐coated and uncoated control grafts, indicating a substantial inhibitory effect on platelet adhesion to the graft surface. NO has a very short half‐life and is continuously scavenged by oxygen and hemoglobin in red blood cells, which averts the cytotoxic effects of any accumulated NO in the local region. The application of NO is not limited to only reducing platelet adhesion. NO also reduces the proliferation and migration of smooth muscle cells and inhibits leukocyte adhesion, which are important factors in the intimal hyperplastic response.^[^ ^242^, ^243^ ^]^

### 4.4 Tumor Targeting

Tumor angiogenesis is an important factor that is commonly considered to be a negative prognostic indicator. This is because angiogenesis increases the blood supply and subsequent delivery of nutrients to the tumor, which facilitates tumor growth.^[^ ^244^ ^]^ Many strategies have been applied to cut down or reduce the blood supply in tumors, such as targeting inhibitory pathways for VEGF which results in the depletion of vascular structures and a decreased blood supply to tumors.^[^ ^245^ ^]^ However, recent studies revealed that strategies to reduce the blood supply to tumors result in drug resistance, tumor hypoxia, and metastasis.^[^ ^244^, ^245^, ^246^ ^]^ Counterintuitively, improved blood flow in tumor‐specific regions through angiogenesis and blood vessel dilation showed better drug delivery as well as improved therapeutic outcomes.^[^ ^244^, ^247^ ^]^ The reports showed that increased blood flow in hypoxic regions of tumors increases the susceptibility of those cells to radiotherapy treatments.^[^ ^159^, ^248^ ^]^ The potential role of NO as part of tumor treatments has recently been studied due to its selective cytotoxic effects on tumor‐specific cells. But as with previously mentioned therapeutic applications of NO, one of the major hurdles to overcome is the short half‐life of endogenously generated NO. To circumvent this issue, targeted NO donors are used to specifically induce an anti‐tumor effect through its pro‐apoptotic cytotoxicity. The formation of toxic and mutagenic superoxide species, like ONOO^−^ and N_2_O_3_, at the site of a tumor induces a pro‐apoptotic effect through DNA damage, gene mutation, protein inactivation, and inhibition of DNA repair mechanisms.^[^ ^138^, ^159^, ^249^ ^]^ The efficacies of anti‐cancer drugs (e.g., Paclitaxel (PTX) and Doxorubicin) have been studied as part of cancer treatments, and in some cases, drug resistance has been observed. Therefore, to improve the cytotoxic effects that these drugs have on cancer cells, as well as to decrease the occurrence of drug resistance, NO donor‐based systems were co‐administered with these drugs. Recent studies showed that these types of NO combined systems exhibit improved levels of efficacy, decreased levels of multidrug resistance, and decrease in cancer metastasis.^[^ ^244^, ^250^ ^]^

**Figure 10:**
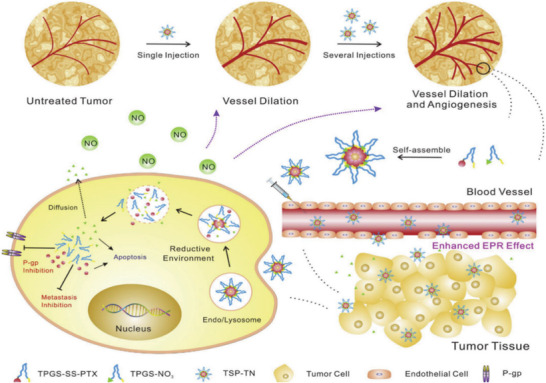
Schematic illustration of the enhanced tumor therapy with reversed multidrug resistance (MDR) tumor cells and inhibited metastasis by drug co‐delivery and in situ vascular‐promoting strategy. The compounds under test are d‐α‐tocopherol polyethylene 1000 glycol succinate (TPGS), TPGS‐paclitaxel (TPGS‐SS‐PTX or TSP), TPGS derived NO donor (TPGS‐NO_3_ or TN), and micellar TPGS‐SS‐PTX and TPGS‐NO_3_ (TSP‐TN). TSP‐TN not only caused vasodilation and angiogenesis reducing leaky vasculature but also induced apoptosis in MDR tumor cells. Reproduced with permission.^[^ ^244b^ ^]^ Copyright 2017, Elsevier Inc.

**Figure 11:**
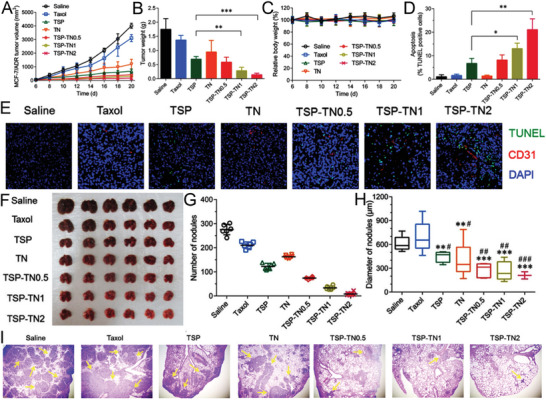
In vivo antitumor efficacy against MDR tumors. A) Change in tumor (MCF‐7/ADR) volume profile, B) Tumor weight, C) Relative body weight, and D) Tunnel assay to evaluate apoptosis of tumor cells in mice *p < 0.05, **p < 0.01, and ***p < 0.001 versus TSP. E) Blood vessels (α‐CD31 antibody stained as red and DAPI (blue) for nuclei) and tumor apoptosis (TUNEL staining as green) presented by immune‐fluorescent images. F) Digital images of dissected lungs in B16F10 metastatic model. G,H) Number and diameter of nodules in metastatic lungs. *p < 0.05, **p < 0.01, and ***p < 0.001 versus saline. #p < 0.05, ##p < 0.01, and ###p < 0.001 versus Taxol. I) H&E images of metastatic lung sections. Reproduced with permission.^[^ ^244b^ ^]^ Copyright 2017, Elsevier Inc.

Yin et al. prepared an anti‐cancer drug model based on the usage of PTX along with a NO donor stabilized polymeric system involving d‐α‐tocopherol polyethylene 1000 glycol succinate (TPGS), TPGS‐TN.^[^ ^244b^ ^]^ The TPGS‐NO_3_ that dissociated from TSP‐TN promoted the drug delivery of PTX and inhibited tumor growth by increasing vessel dilation in the tumor tissue. The synergistic antitumor effect of PTX and NO can potentially increase angiogenesis and vessel dilation for several anticancer therapies without causing drug resistance in the involved pathway (**Figure** **10**). The anti‐tumor effect of different concentrations of TSP‐TN was compared to Taxol and TSP as a part of an in vivo study involving drug‐resistant MCF‐7/ADR tumor‐bearing mice. (**Figure** **11**).^[^ ^244b^ ^]^ The volume and weight of the tumors decreased significantly in the cases involving different concentrations of TSP‐TN, but no significant change was observed in the cases of the control, Taxol, and TSP groups (Figure 11A,B). Further, the body weight of the mice treated with TSP‐TN remained almost the same as that of the mice in the control group, which demonstrated the tumor‐specific activity of TSP‐TN (Figure 11C). The dose‐dependent anti‐tumor effect of TSP‐TN showed to induce massive amounts of apoptosis of the tumor cells in contrast to the control, Taxol, TSP, and TSN groups (Figure 11D,E). The immune‐fluorescent images indicated an increase in the signaling of apoptotic cells and blood vessels in TSP‐TN groups, and low or no signaling was observed in the other groups (Figure 11E). NO can also restrict tumor metastasis when evaluated in the B16F10 metastatic tumor model, confirmed by the decreased number and diameter of nodules (Figure 11F–H). The substantial tumor‐inhibiting effect was further asserted by H and E staining of metastatic lung sections (Figure 11I). The application of exogenous NO donors along with photothermal chemotherapy is a novel approach to cancer therapy reported by Du et al. The study used a d‐α‐tocopherol polyethylene 1000 glycol succinate‐galactose‐based system for loading NO donors with Doxorubicin (NO‐DOX@PDA‐TPGS‐Gal) and tested them against drug‐resistant hepatocellular carcinoma (HCC) induced subcutaneously in BALB/c mice.^[^ ^250^ ^]^ When the NPs were applied to the tumor and exposed to NIR, they responded by generating heat within the tumor and releasing NO in micromolar quantities. When tested in vivo, the combined approach of using NO‐DOX@PDA‐TPGS‐Gal along with NIR exhibited the potential to completely suppress tumor growth and reduce the tumor size over a period of 21 days.

NO, in micromolar concentrations, can induce apoptosis and assist in providing effective anticancer treatment.^[^ ^178^ ^]^ Polymeric nano delivery systems consisting of poly‐(6‐O methacryloyl‐d‐galactose) based NONOate‐multiarm can generate NO in micromolar concentrations to achieve desired therapeutic effects.^[^ ^251^ ^]^ In tumor‐bearing nude mice, in contrast to NO‐donor prodrug JS‐K (O2‐(2, 4‐Dinitrophenyl) 1‐[(4‐ ethoxycarbonyl) piperazin‐1‐yl] diazen‐1ium‐1, 2‐diolate) treated mice, the tumor inhibition and mouse survival rate was substantial in the group treated with the NONOate‐multiarm containing nano delivery system. Such systems have shown the capability of increasing the half‐life of NO by up to 9.2 h, as revealed by kinetic measurements in an in‐vitro system.^[^ ^251^ ^]^ However, in the case of intracranial tumors, delivery of NO and other drugs across the blood‐brain barrier (BBB) has always been a major challenge. Although there are a few studies that demonstrate efficient delivery of NO across the BBB, in the case of targeting and treating intracranial tumors, without adequate tissue penetration it is unfeasible to employ an external stimulus with the precision necessary. In recent years, the diagnostic and therapeutic advantages of implantable optoelectronic devices have attracted the focus of the scientific community because of their potential to serve as powerful medical tools. For example, a carmustine‐delivering implantable device for the treatment of neuroglioma has been clinically approved in the form of a carmustine wafer implant (Gliadel wafer) for the controlled and prolonged release of carmustine.^[^ ^252^ ^]^ Yao et al. developed an integrated implantable NO‐generating device for treating intracranial neuroglioma that can self‐power and regulate the release of NO wirelessly.^[^ ^253^ ^]^ The self‐powering ability of the integrated system is comprised of a triboelectric nanogenerator (TENG) and an s‐nitroso glutathione donor encapsulated device for NO generation, which is controlled wirelessly through patients’ smartphones. The TENG generates electrical energy from the patients’ movement, through biomechanical energy conversion, and provides a sustainable power source for the localized release of NO. The efficient therapeutic potential of the self‐powered integrated system was successfully validated in in‐vitro and in‐vivo breast tumors and intracranial tumors as proof of concept.

### 4.5 NO in Other Tissue Engineering and Regenerative Medicine Applications

**Figure 12:**
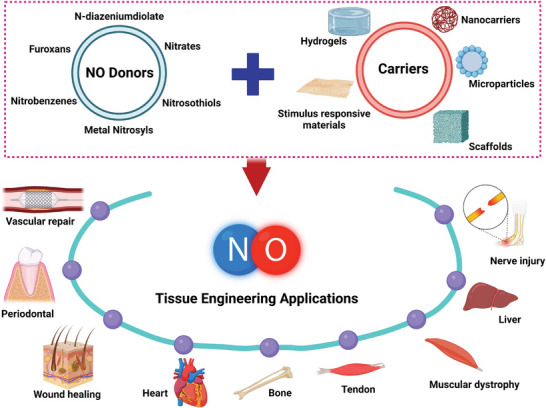
NO delivery for tissue repair and regeneration.

Due to its remarkable multifaceted therapeutic applications, NO has shown great potential in tissue engineering and regenerative medicine (**Figure** **12**). Many reports have revealed the significant roles of NO in skin repair and cardiovascular regeneration. Studies have also exploited the contributions of NO in other regenerative applications (e.g., peripheral nerve regeneration, liver regeneration, endothelium regeneration, bone regeneration, and muscle regeneration).^[^ ^254^ ^]^

**Figure 13:**
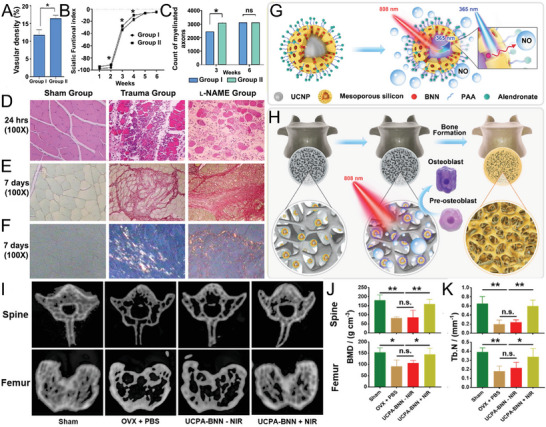
NO promotes the regeneration of nerves, muscle, and bone. A–C) Quantitative data showing enhanced revascularization, motor functioning, and nerve regeneration respectively, with NO‐SN in nerve crush injury rat model. Reproduced with permission under Creative Commons Attribution License (CC BY).^[^ ^256^ ^]^ Copyright 2021, The Authors. Published by Wolters Kluwer. D) H&E staining of muscle sections from the gastrocnemius injury site showed inflammatory and more fibrotic tissue formation in _L_‐NAME treated group (iNOS inhibitor) after 24 h compared to sham and trauma groups. E,F) Increased collagen deposition in _L_‐NAME treated group after 7 days as compared to other groups showed by Sirius red staining alone (E) and under polarized light (F). Reproduced with permission.^[^ ^259a^ ^]^ Copyright 2010, Elsevier Inc. Panels (G,H) show the scheme of Osteoporosis treatment using NIR‐regulated UCPA‐BNN gas therapy in the OVX model. Panel (I) shows the microarchitecture of trabecular bone using micro‐CT for the third lumbar vertebral body and the distal femur of each group. Panels (J) and (K) represent the quantitative data of architectural parameters of BMD (bone mineral density) and Tb. N (trabecular number) respectively, in OVX mice. Reprinted with permission.^[^ ^266^ ^]^ Copyright 2021, American Chemical Society.

Peripheral nerve injury (PNI), an intricate histopathological process, is a grave clinical concern that leads to axonal degeneration or demyelination and subsequent muscle weakness and loss of motor/sensory functions. The recovery of nerve function is challenging due to the slower regenerative capability of peripheral nerves, and regenerative outcomes are comparatively poor.^[^ ^255^ ^]^ To date, a few reports have suggested that peripheral nerve regeneration and degeneration are modulated by NO release produced by iNOS and nNOS.^[^ ^256^ ^]^ The NO released by these critical factors plays a key role in peripheral nerve repair and regeneration.^[^ ^257^ ^]^ The NO released due to the upregulation of iNOS in PNI causes local vasodilation and increased blood flow to the nerve which may help in the removal of myelin and axonal debris.^[^ ^256^ ^]^ The substantial effect of NO on nerve regeneration was reported earlier in an iNOS‐lacking mouse model.^[^ ^254b^ ^]^ The study showed slow myelin degeneration and in turn delayed myelin fiber regeneration, as well as the promotion of delayed neuropathic pain. In PNI, to promote axonal regeneration, regulating NO concentrations during the neural regeneration process is highly recommended. Lee et al. recently reported improved functional recovery in NO‐treated animals through the mechanism of boosted axonal regeneration. The study developed NO‐silica NPs (NO‐SN) incorporated in fibrin glue and validated its potential therapeutic role in enhancing revascularization and nerve regeneration in an acute peripheral nerve crush injury model (**Figure** **13**A–C). High vascular density was observed in NO‐SN treated group after 3 days of nerve crush injury as compared to the untreated group (Figure 13A). Overall, increased sciatic nerve functional recovery and fast nerve regeneration after 2 and 3 weeks respectively was observed in the NO‐treated group than in the control group, indicating an early functional recovery in the early phase (Figure 13B,C).^[^ ^256^ ^]^

Muscle regeneration and function are severely affected by post‐traumatic muscle injuries due to improper and slow healing. Additionally, this type of improper healing is usually associated with scar tissue formation and increased chances of recurrent injuries.^[^ ^258^ ^]^ For successful muscle repair and functional recovery, activation of quiescent satellite cells, infiltration of inflammatory cells, and maintenance of the balance between the processes of regeneration and fibrosis are all prerequisites.^[^ ^254^, ^259^ ^]^ The regeneration and homeostasis of adult skeletal muscle are improved by NO signaling which promotes satellite cell activation, proliferation, migration, angiogenesis, and endorses macrophage activity.^[^ ^174^, ^260^ ^]^ Rigamonti et al. were the first to elucidate that during a muscle injury, elevated iNOS expression is associated with macrophages and promotes muscle regeneration.^[^ ^254f^ ^]^ Recently, in another study, SIN‐1 (an NO donor) triggered myoblast differentiation, thereby promoting myogenesis. The authors inferred that elevated NO levels had substantial contributions to the process of skeletal muscle regeneration and were believed to be its central modulator.^[^ ^261^ ^]^ Further, in a crush muscle injury model, early phase NO production was found to be a key factor in the repair of skeletal muscle.^[^ ^259a^ ^]^ In the early phases of crush muscle injury, blocking of NO production in _L_‐NAME (NOS inhibitor)‐treated group resulted in inflammation and edema after 24 h and poor muscle regeneration and formation of more fibrotic scar post 7 days trauma in contrast to the sham group (Figure 13D–F).

Endothelium regeneration has a critical role in improving vascularization and perfusion during healing and the process of tissue regeneration. In the case of pathological diseases that mainly cause severe disruption of the endothelium of vessels, different types of grafts or implants are used to mimic the native vessel or to regenerate the vascular tissue and recapitulate its biological functions.^[^ ^262^ ^]^ However, challenges like tissue integration, low vessel patency, risk of infections, clotting, etc. persist and limit their translational potential.^[^ ^263^ ^]^ Hence, it is important to consider the structure, components, and functions of vessels, in addition to the choice of materials used when designing and fabricating advanced grafts.^[^ ^262^, ^263^ ^]^ As discussed earlier, NO is endogenously released by endothelial cells, which line the inner wall of the vessels, to maintain vascular health. NO boosts migration of endothelial cells, promotes angiogenesis, regulates vascular patency, and improves vascular tissue regeneration. Duo et al. demonstrated the potential application of NO‐generating PCL/sulfonated keratin mats in vascular tissue regeneration by showing improved cell viability, low platelet adhesion, and enhanced re‐endothelialization.^[^ ^264^ ^]^

Apart from that, many researchers have explored the role of endogenous and exogenous NO in bone regeneration, demonstrating that NO has a remarkable contribution to osteogenesis‐related physiological and pathological conditions.^[^ ^254^, ^265^ ^]^ In addition, multiple studies suggested that NO modulates bone cell differentiation, proliferation, and survival during bone metabolism. It also displays the ability to contribute considerably to the process of bone healing in cases of fractures when used in combination with bone morphogenic protein.^[^ ^254^, ^265^ ^]^ In a recent study, the targeted release of NO from a BNN precursor using NIR‐sensitive upconversion nanoparticles (UCNP) coated mesoporous silica NPs was used for targeted bone formation (Figure 13G,H). In contrast to control groups, the effect of NO release from the UCPA‐BNN + NIR group on osteoporosis in ovariectomized‐induced mice potentially showed substantial osteogenesis and improved recovery in bone mass as observed from micro CT, followed by its quantitative analysis (Figure 13I–K).^[^ ^266^ ^]^ The potential ability to avoid or even reverse osteoporosis is primarily due to the effective and targeted release of NO.

Other studies have reported a significant contribution from NO in pulp‐dentin regeneration, tendon regeneration, and liver regeneration.^[^ ^173^, ^254^, ^267^ ^]^ However, advanced and comprehensive research in this direction is necessary to further explore the role of NO in these tissue engineering applications.

## 5 Conclusions and Perspectives

In summary, we have reviewed the remarkable roles of NO in biological systems and the use of NO as a therapeutic agent in different biomedical applications. We have also highlighted the use of distinct NO donors/carriers for efficiently generating NO or sustained and prolonged release of NO at target sites. In the last decade, the therapeutic roles of NO have gained considerable attention from the scientific community, specifically regarding the exogenous delivery of NO using NO donors/carriers. However, these NO donors/carriers are associated with major challenges, such as the regulation of NO payloads, short half‐lives, toxicity, duration of NO release, etc. Addressing these issues requires a thorough physicochemical characterization and effective ways to establish a stable system for their actual use in controlled NO delivery at targeted sites. This presentation focused on substantiating state‐of‐the‐art advancements and strategies in overcoming the challenges associated with the biological administration of NO, including a review of the fundamental biological roles of NO, and diverse approaches that have been employed in the design and development of desired NO donors/carriers for distinctive biomedical applications.

The NO released from different types of polymeric particles, liposomes, and other polymeric implantable donors/carriers has proved effective in accomplishing the potential therapeutic effects of NO in various pathological conditions. However, further advancement in the design and synthesis of NO‐releasing systems is required to properly regulate the concentration, delivery, and toxicity of NO. To these ends, multifunctional nano/micro or MOF‐based NO delivery vehicles can facilitate the therapeutic applications of NO. The development of stimuli‐responsive and functionalized nanomaterial for NO delivery would provide a promising platform for achieving significant bactericidal effects, enhancement of chemotherapeutic effects in treating tumors, and numerous other NO‐based biomedical modulatory effects. A new and emerging approach for the efficient application of NO effects involves the possibility of on‐site NO generation in a controlled manner. Moreover, many studies have demonstrated the critical role of NO in tissue repair and regeneration. However, the potential therapeutic application of NO in this domain is still primarily unexplored. Even though a plethora of studies have elucidated the effective role of NO in different pathological conditions, the focus needs to shift to evaluating the stability, biodistribution, and toxicity of NO over extended periods as part of in‐vivo studies, which may aid in its translational applications.^[^ ^163c^ ^]^

## Conflict of Interest

The authors declare no conflict of interest.
